# Studying SARS-CoV-2 interactions using phage-displayed receptor binding domain as a model protein

**DOI:** 10.1038/s41598-023-50450-4

**Published:** 2024-01-06

**Authors:** Beatriz Pérez-Massón, Yazmina Quintana-Pérez, Yaima Tundidor, Dayana Pérez-Martínez, Camila Castro-Martínez, Mario Pupo-Meriño, Ivette Orosa, Ernesto Relova-Hernández, Rosmery Villegas, Osmany Guirola, Gertrudis Rojas

**Affiliations:** 1 Center of Molecular Immunology, Calle 216 esq 15, apartado 16040, Atabey, Playa, CP 11300 Havana, Cuba; 2 Universidad de Ciencias Informáticas, Carretera a San Antonio de los Baños, km 2 1/2, Torrens, Boyeros, CP 19370 Havana, Cuba; 3 Center for Genetic Engineering and Biotechnology, Ave 31 E/158 y 190, Cubanacán, Playa, CP 11300 Havana, Cuba

**Keywords:** Biological techniques, Biotechnology, Computational biology and bioinformatics, Immunology, Molecular biology

## Abstract

SARS-CoV-2 receptor binding domain (RBD) mediates viral entry into human cells through its interaction with angiotensin converting enzyme 2 (ACE2). Most neutralizing antibodies elicited by infection or vaccination target this domain. Such a functional relevance, together with large RBD sequence variability arising during viral spreading, point to the need of exploring the complex landscape of interactions between RBD-derived variants, ACE2 and antibodies. The current work was aimed at developing a simple platform to do so. Biologically active and antigenic Wuhan-Hu-1 RBD, as well as mutated RBD variants found in nature, were successfully displayed on filamentous phages. Mutational scanning confirmed the global plasticity of the receptor binding motif within RBD, highlighted residues playing a critical role in receptor binding, and identified mutations strengthening the interaction. The ability of vaccine-induced antibodies to inhibit ACE2 binding of many mutated RBD variants, albeit at different extents, was shown. Amino acid replacements which could compromise such inhibitory potential were underscored. The expansion of our approach could be the starting point for a large-scale phage-based exploration of diversity within RBD of SARS-CoV-2 and related coronaviruses, useful to understand structure–function relationships, to engineer RBD proteins, and to anticipate changes to watch during viral evolution.

## Introduction

Severe Acute Respiratory Syndrome Coronavirus 2 (SARS-CoV-2) is the pathogen responsible for Coronavirus disease pandemic started in 2019 (COVID-19)^1,2^. The viral particle includes four structural proteins: membrane (M), nucleocapsid (N), envelope (E) and spike (S)^3^. The S protein has two subunits: S1 which contains the receptor binding domain (RBD) and the membrane anchored S2 subunit able to mediate the fusion between virus and host cell membranes^4^. RBD binding to angiotensin converting enzyme 2 (ACE2), the receptor on human cells, is the first event in the cascade leading to viral entry^1,4,5^. Blocking this interaction is the rationale behind the development of therapeutic antibodies and vaccines to treat/prevent COVID-19. Several anti-SARS-CoV-2 vaccines with proved efficacy in the prevention of severe disease and death have thus been developed^6–10^, using the spike protein or just the RBD as antigen. The RBD spans from residue 331–524 of S protein and includes amino acids (aa) in direct contact with ACE2, concentrated in the so-called receptor binding motif (RBM)^11,12^. SARS-CoV-2 RBD is an immunodominant region and the target of most neutralizing antibodies induced by the viral infection^13,14^ and vaccination^15^.

Since the beginning of the pandemic, new SARS-CoV-2 variants have risen and rapidly spread across the world, challenging public health systems. Emerging mutations confer increased transmissibility, higher virulence or the ability to escape from neutralization by antibodies. These features have been considered by the World Health Organization (WHO) to define five major variants of concern (VOC) currently known as Alpha (B.1.1.7, December 2020)^16^, Beta (B.1.351, December 2020)^17^, Gamma (P.1, January 2021)^18^, Delta (B.1.617.2, May 2021)^19^, and more recently the Omicron family (B.1.1.529, November 2021)^20^ which now comprises several sub-variants. A broader class includes multiple viral variants of interest (VOI), detected and monitored along the pandemic. Part of viral diversity resides in RBD, where replacements can cause increased receptor binding affinity and/or escape from neutralizing antibodies^21,22^. Both forces play a role in driving viral evolution^23^. High throughput screening of RBD interactions is thus relevant in two scenarios: understanding changes already found in nature and predicting the impact of further variations. Several platforms have been used to this end. Complete maps of the effects of mutations on RBD stability and receptor binding^24^, and recognition by monoclonal antibodies (mAbs)^25^ or polyclonal convalescent plasma^26^, were generated using yeast display and deep mutational scanning. A similar methodology was used to identify RBD variants that escape from antibody neutralization^27^. These approaches combined the display of a properly folded antigen on eukaryotic host cells with the ability to screen millions of variants in a quick and automation-amenable manner.

The current work validates the use phage-displayed RBD as a model to explore RBD interactions with ACE2 receptor, mAbs and polyclonal antisera. Biologically active and antigenic SARS-CoV-2 Wuhan-Hu-1 RBD, as well as mutated RBD versions from viral VOC, have been displayed on filamentous phage. Mutational scanning in this format allowed direct evaluation of ACE2 binding properties of hundreds of single-mutated variants, resulting in the identification of a few critical residues and highlighting the global plasticity of the receptor binding surface. The implications of individual replacements for escape from pre-existing neutralizing antibodies elicited by vaccination were also explored, showing susceptibility of all tested single-mutated variants to inhibition by human polyclonal antibodies, albeit at different degrees. The intrinsic simplicity of phage display (the oldest and most extended protein display platform^28^) makes our approach accessible to any laboratory with basic molecular biology facilities. High throughput potential of phage display, linked to the construction of large libraries and their screening through DNA deep sequencing technologies, could allow large scale simulations of RBD molecular evolution, shedding new light on our understanding of the already observed viral changes, but also predicting possible future events that need to be carefully watched.

## Results

### Phage-displayed SARS-CoV-2 RBD kept the antigenicity and biological activity of its natural counterpart

**Figure 1:**
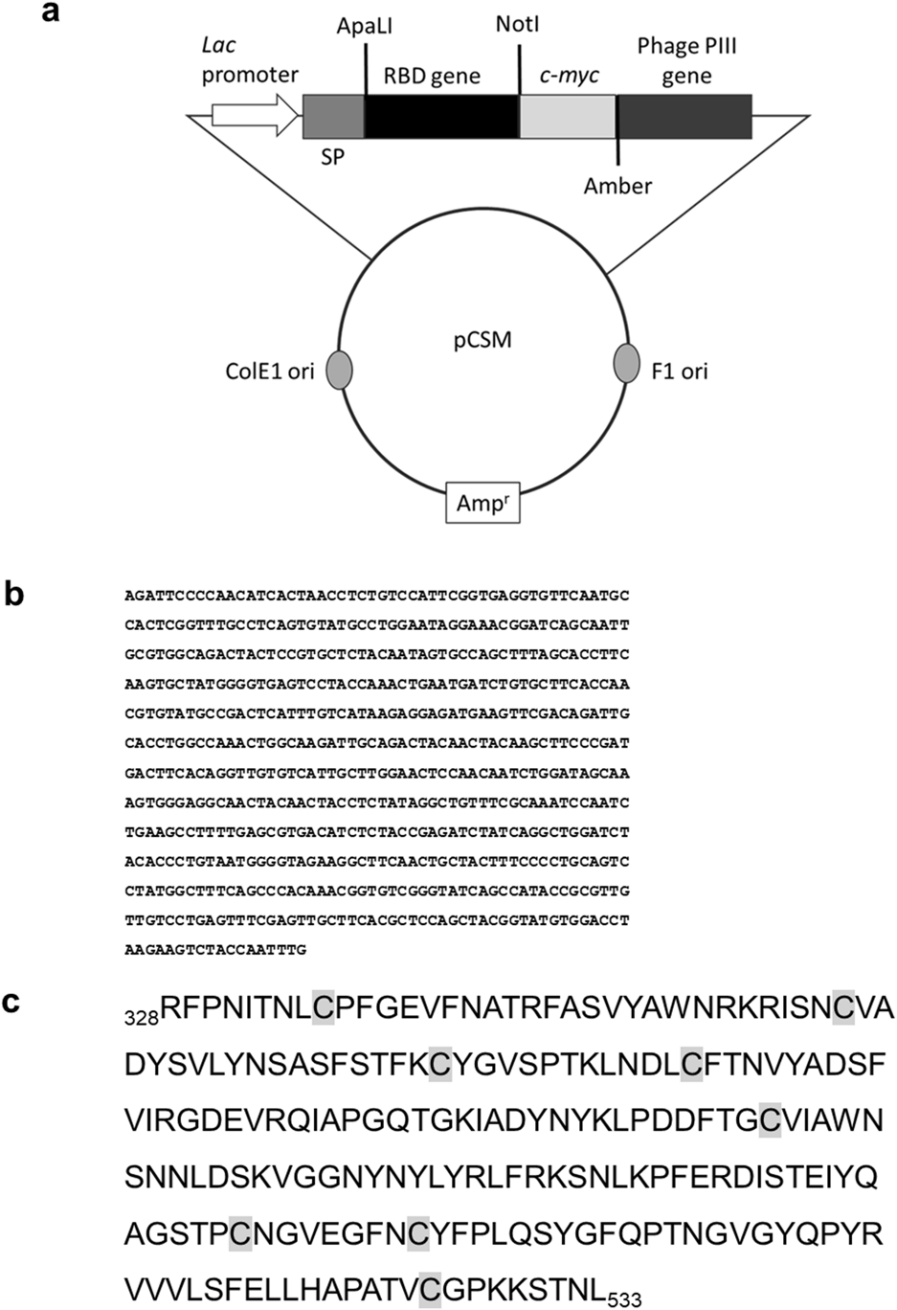
Schematic representation of the genetic construct used for RBD phage display (**a**). pCSM phagemid vector includes the *Lac* promoter, an ampicillin resistance gene, as well as phage and *E.* *coli* replication origins. It also contains the genes encoding DsbA signal peptide, *c-myc* tag and M13 PIII protein. An amber stop codon is located between tag and PIII genes. The gene coding for Wuhan-Hu-1 RBD (fragment 328–533 of Spike protein, **b**) was cloned between ApaLI and NotI restriction sites. Deduced amino acid sequence of the displayed RBD is shown (**c**). Cysteine residues are shaded in grey.

**Figure 2:**
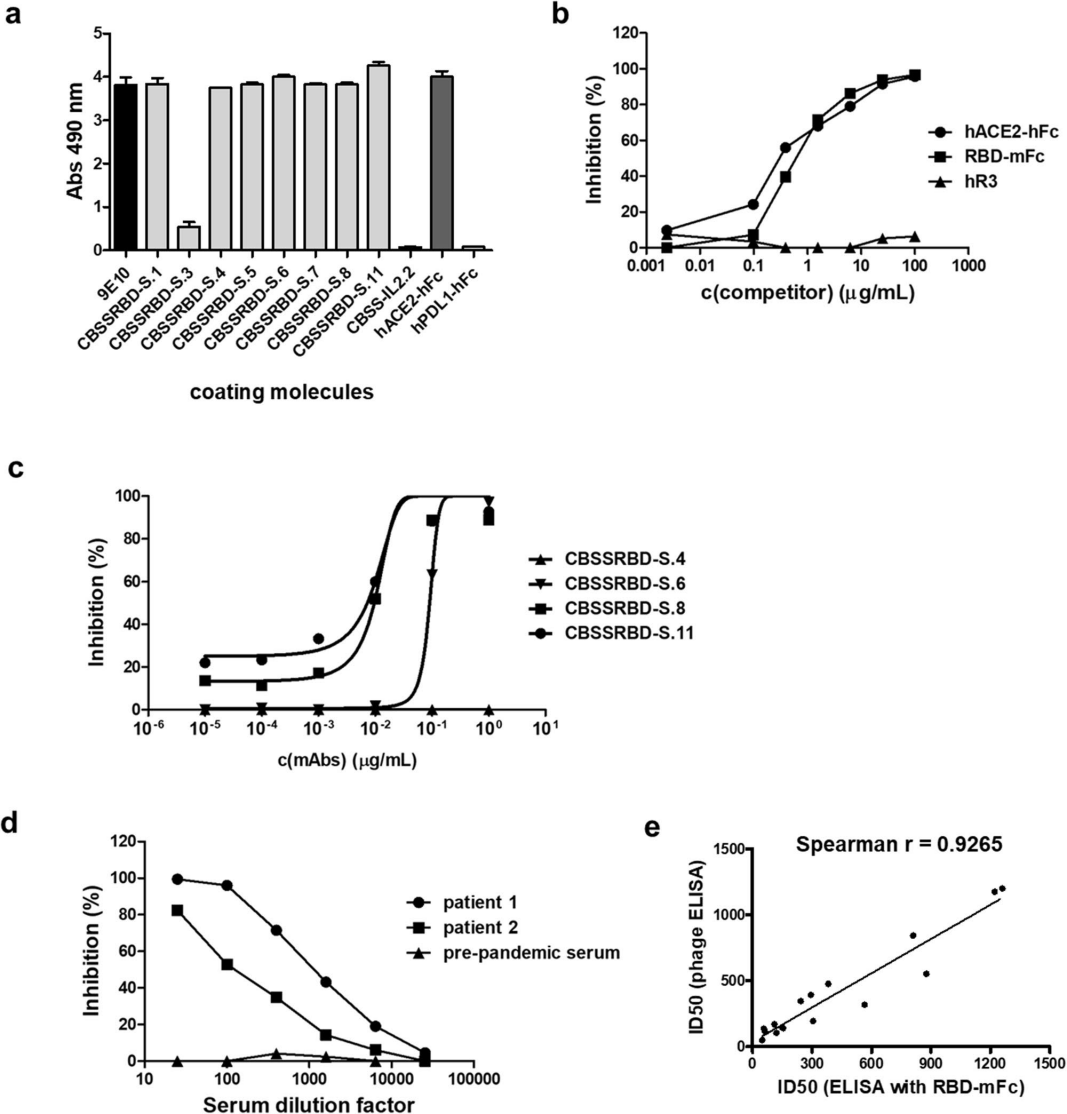
Antigenicity and biological activity of phage-displayed Wuhan-Hu-1 RBD. Phages displaying RBD (10^11^ viral particles/mL) were incubated on polyvinyl chloride microplates coated with the anti-*c-myc* tag antibody 9E10, anti-RBD mAbs (CBSSRBD-S.1-CBSSRBD-S.11), and a recombinant protein comprising human ACE2 extracellular domain (ECD) fused to human IgG1 Fc (hACE2-hFc). Anti-IL2 CBSS-IL2.2 mAb and the fusion protein hPDL1-hFc were used as unrelated coating molecules to assess non-specific background levels. Bound phages were detected with an anti-M13 PVIII mAb conjugated to horseradish peroxidase (**a**). Binding of phage-displayed RBD to immobilized hACE2-hFc was inhibited by two soluble recombinant fusion proteins: hACE2-hFc itself and a second protein comprising RBD fused to mouse IgG2a (RBD-mFc) (**b**), and not by a humanized antibody targeting EGF Receptor (hR3) included as negative control. Inhibition by neutralizing anti-RBD antibodies CBSSRBD-S.6, CBSSRBD-S.8 and CBSSRBD-S.11 (**c**) and by sera of COVID-19 convalescent patients (**d**) was also shown. Non-neutralizing CBSSRBD-S.4 mAb and negative pre-pandemic serum were used as negative controls in each case. Sera from sixteen COVID-19 convalescent patients were evaluated by the above-described competition assay and in a similar experiment using RBD fused to mouse IgG2a Fc (RBD-mFc) as the probe and anti-mouse IgG conjugated to HRP for detection. Half-maximal inhibitory dilution (ID50) for every sample in each assay was determined after fitting the data to sigmoidal inhibition curves. Spearman correlation was used to determine the association between the ID50 values obtained in both formats (**e**). A significant correlation was observed (*p *< 0.0001).

Wuhan-Hu-1 SARS-CoV-2 S protein fragment 328–533 (comprising the minimal RBD^11^ plus short extensions at both N- and C- termini) was successfully displayed on filamentous phage using a PIII-fusion phagemid-based system (Fig. 1). Eight cysteine residues, able to form the four disulfide bonds that keep RBD architecture, were included in the displayed protein. The presence of RBD on phage surface was detected by enzyme-linked immunosorbent assay (ELISA) using 9E10, a mAb against the *c-myc* tag inserted between the displayed protein and M13 PIII, as capture molecule (Fig. 2a). Recognition of RBD itself by a panel of mAbs raised against recombinant RBD produced in human host cells^29^ showed that the phage-displayed version keeps the antigenicity of its soluble counterpart (Fig. 2a). Since all but one of these anti-RBD antibodies bind conformational epitopes, as shown by the total or partial loss of reactivity upon disulfide bond reducing treatment (Suppl. Fig. S1), their ability to capture phage particles was a proof of the proper folding of displayed RBD. The weak reactivity of CBSSRBD-S.3 mAb compared to the rest (Fig. 2a) did not imply a folding defect, as this antibody is the only one that recognizes a linear epitope and its reactivity was even increased upon antigen denaturation (Suppl. Fig. S1).

Biological activity of phage-displayed RBD was shown by the ability to bind its natural interacting partner, ACE2 receptor extracellular domain (ECD) (Fig. 2a). The specificity of this interaction was confirmed using recombinant versions of either RBD or ACE2 ECD as competitors in solution, resulting in dose-dependent inhibition (Fig. 2b). Binding was also inhibited by three anti-RBD mAbs (CBSSRBD-S.6, CBSSRBD-S.8 and CBSSRBD-S.11) (Fig. 2c), previously classified as neutralizing with a cell-based viral neutralization test^29^. The most solid proof of the quality of RBD display was the inhibition of its binding to ACE2 by sera of convalescent patients (Fig. 2d), showing the antigenic equivalency between the phage-displayed version and the natural domain on SARS-CoV-2, as antibodies in sera had been elicited by the virus itself during infection (prior to vaccination campaigns).

The above-described results indicated that the first analytical application of phage-displayed RBD could be as a probe to evaluate inhibitory capacity of antibodies elicited by infection and/or vaccination. A broader panel of COVID-19 convalescent patients’ sera was tested. All sera, with the exception of the pre-pandemic serum used as negative control, inhibited binding of phage-displayed RBD to ACE2 (Suppl. Fig. S2). Sera were analyzed in parallel by a similar assay using recombinant RBD-mouse Fc fusion protein as probe. The latter had been used to characterize humoral responses during vaccine development and clinical trials^30^. Half-maximal inhibitory dilution (ID50) was calculated for each serum sample using both assays. Even though the absolute ID50 values were dependent on the assay format, there was a strong positive correlation between the results of both experiments (Fig. 2e), validating the usefulness of the phage-based assay to characterize anti-RBD antibodies in terms of receptor binding inhibitory activity.

### Phage display allowed characterization of RBDs from SARS-CoV-2 variants of concern

**Figure 3:**
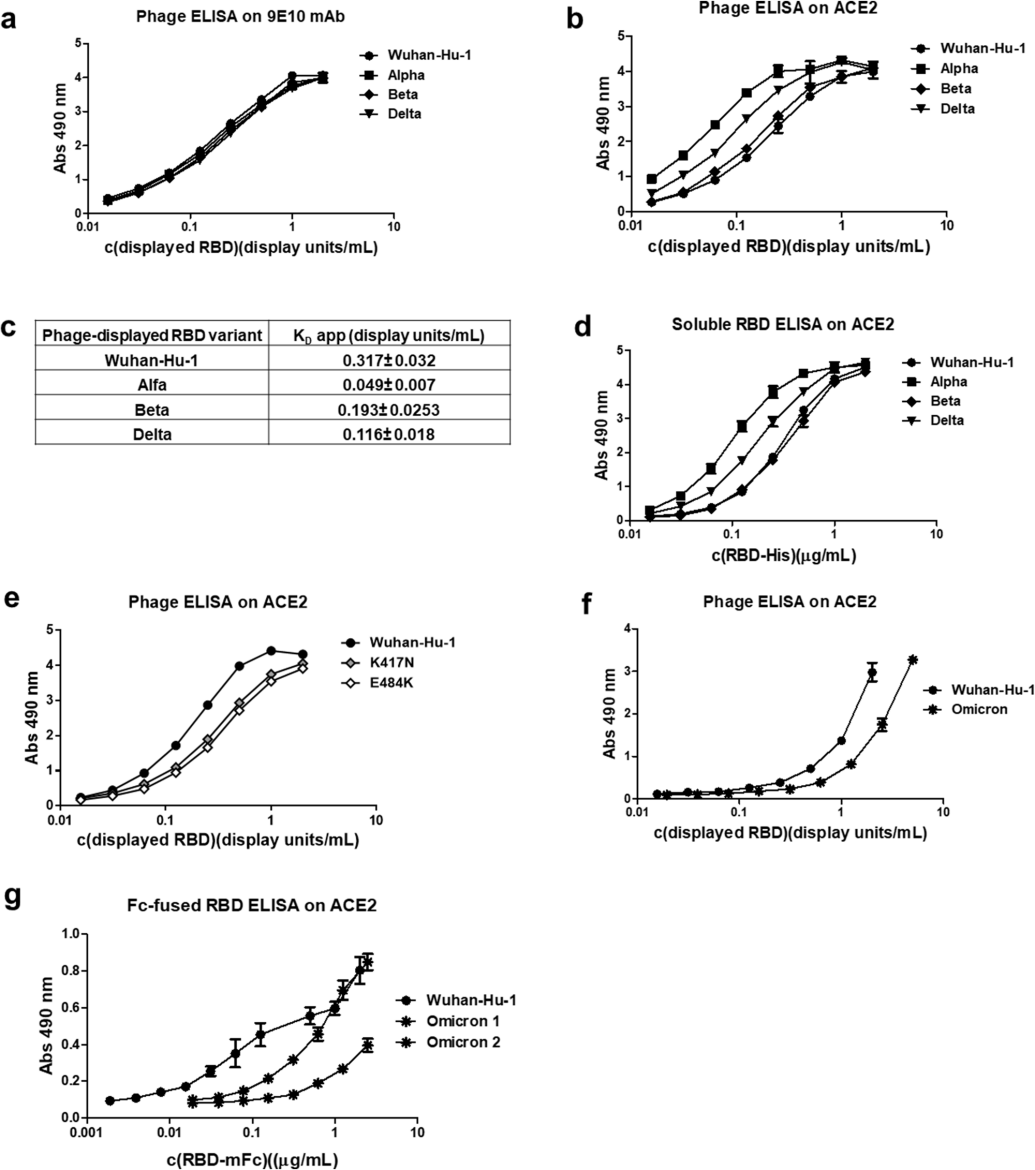
Characterization of mutated RBD from viral variants of concern. Equivalent amounts of phage-displayed RBD variants (Alpha, Beta and Delta) were incubated on polyvinyl chloride microplates coated with either the anti-tag 9E10 mAb (**a**), or a recombinant protein comprising the extracellular domain of human ACE2 fused to a human IgG1 Fc domain (hACE2-hFc) (**b**). Phage-displayed Wuhan-Hu-1 RBD was included as reference. Bound phages were detected with an anti-M13 PVIII mAb conjugated to horseradish peroxidase (HRP). Data from ELISA on ACE2 were fitted to binding-saturation curves to determine apparent K_D_ values of each variant for the receptor (**c**). The same panel of RBD mutated variants, produced as hexa-His-tagged soluble recombinant proteins by transiently transfected HEK-293 T cells, were evaluated on microplates coated with hACE2-hFc. Bound proteins were detected with an anti-His tag mAb conjugated with HRP (**d**). Single-mutated RBD variants K417N and E484K (**e**), as well as Omicron BA.1 variant (**f**) were evaluated using the above-described phage ELISA. Two preparations of Omicron BA.1 RBD, fused to mouse IgG2a Fc and produced by HEK-293 cells upon stable lentiviral transduction, were also tested on hACE2-hFc-coated microplates together with a similarly formatted fusion protein containing the original Wuhan-Hu-1 RBD. Bound fusion proteins were detected with an anti-mouse IgG antibody conjugated to HRP (**g**).

The simplicity of phage display platform, coupled to site-directed mutagenesis, allowed a fast screening of mutated RBD variants arising during global spreading of the infection. Phage-displayed mutated RBDs corresponding to viral VOC (Alpha, Beta and Delta) kept total or partial antigenicity when probed with several anti-RBD mAbs (Suppl. Fig. S3), excluding gross display artifacts associated to mutations. Normalization of phage preparations according to the display levels (determined through recognition by 9E10 mAb targeting the common *c-myc* tag fused to all of them)^31^ was a pre-requisite for the comparison of biologic activity of mutated RBD versions (Fig. 3a). Assessment of binding ability to ACE2 receptor revealed differences among them. While Alpha and Delta variants exhibited increased ACE2 binding, Beta variant behavior was closer to the original Wuhan-Hu-1 RBD (Fig. 3b). Fitting the data to a binding-saturation equation resulted in a quantitative estimation of the differences, through calculation of apparent K_D_ (K_D_ app) values (Fig. 3c). Phage-displayed Alpha RBD exhibited the highest ACE2 binding ability (more than six-fold increase in comparison to Wuhan-Hu-1 RBD). The same ranking of binding activities was obtained with recombinant His-tagged proteins corresponding to RBD variants produced by transient transfection of human cells (HEK-293 T adapted to grow in suspension) (Fig. 3d), validating the usefulness of phage platform for mutational scanning of the viral antigen.

The fact that binding ability of Beta RBD was similar to the one of the original Wuhan-Hu-1 domain was somehow intriguing, due to the presence of N501Y replacement, which caused the greatly stronger binding of the Alpha variant. Dissecting the effects of the other mutations contained in Beta RBD (K417N and E484K) provided a reasonable explanation, as both replacements decreased binding (Fig. 3e). Their detrimental effects were compensated by N501Y, resulting in conservation of ACE2 binding in the triple-mutated Beta RBD.

### The accumulation of mutations in Omicron RBD resulted in a different behavior of this domain as compared to the other variants

Unlike Alpha, Beta, and Delta RBDs, Omicron BA.1 phage-displayed RBD showed highly variable and substantially decreased display levels: from 5 to 78% (average 29.5%) in comparison with Wuhan-Hu-1 RBD, and a remarkable low ACE2 binding ability after normalization (Fig. 3f), which was unexpected taking into account previous affinity measurements^32^. Reduced display has been previously associated to improper folding and aggregation propensity^33,34^. The fact that phage-displayed Omicron-RBD is still biologically active at high concentrations and reactive with several anti-RBD mAbs (Suppl. Fig. S3) supported its potential usefulness as starting point for further engineering. Total lack of reactivity against CBSSRBD-S.8 and CBSSRBD-S.11 most likely results from specific disruption of their epitopes due to mutations.

Testing Omicron BA.1 RBD through production in the HEK-293 T-based transient transfection system was impossible due to the lack of expression of this variant (Suppl. Fig. S4), which contrasted with successful production of Wuhan-Hu-1 and other VOC RBDs. We were able to produce BA.1 RBD as a mouse IgG2a Fc fusion protein after stable lentiviral transduction of HEK-293 cells. Its characterization revealed a drastically reduced and highly variable ACE2 binding ability (Fig. 3g), and a remarkable aggregation propensity (Suppl. Fig. S5). These features confirmed previous findings with the phage-displayed version and differed from the robustness and homogeneity of a similar fusion protein based on Wuhan-Hu-1 RBD. Taken together, the above-described results pointed to the classification of Omicron BA.1 RBD as a challenging difficult-to-express protein in both mammalian cell expression and phage display scenarios.

### Mutational scanning of phage-displayed RBD revealed a remarkable global plasticity of RBD/ACE2 interface and underscored individually critical residues

Site-directed randomization of selected positions within the RBM of Wuhan Hu-1 SARS-CoV-2 through Kunkel mutagenesis^35^, coupled to phage ELISA screening, was useful to assess directly the effects of single mutations on ACE2 binding. Three segments within RBM were initially defined for targeting (aa 443–456, 473–479, 483–505), comprising multiple residues separated by less than 8 Å of the interacting partner ACE2 and/or previously identified as crucial positions for binding to either ACE2 or anti-RBD antibodies during structural, mutagenesis, or phylogenetic studies. 25 of these positions were individually explored by mutagenesis, including some already postulated to contribute either to the original interaction or to its enhancement when mutated (449, 452, 453, 455, 456, 470, 474, 475, 477,484, 486, 487, 489, 490, 493, 498, 500, 501, 503, 505)^12,24,36–38^. Positions that changed in the emerging viral variants already described by the time when the current work was performed, being members of the former group or not, were also explored (452, 453, 477, 478, 484, 501)^16–19,38,39^. Additional positions were chosen mainly because of their involvement in the formation of epitopes recognized by neutralizing antibodies (445, 473, 483, 494)^26,36^. K417 was the only targeted residue outside the RBM, due to its postulated role in binding and its variability among viral variants^12^. Clones harboring 242 single-mutations were characterized, representing 49% coverage of the explored sequence space.

**Figure 4:**
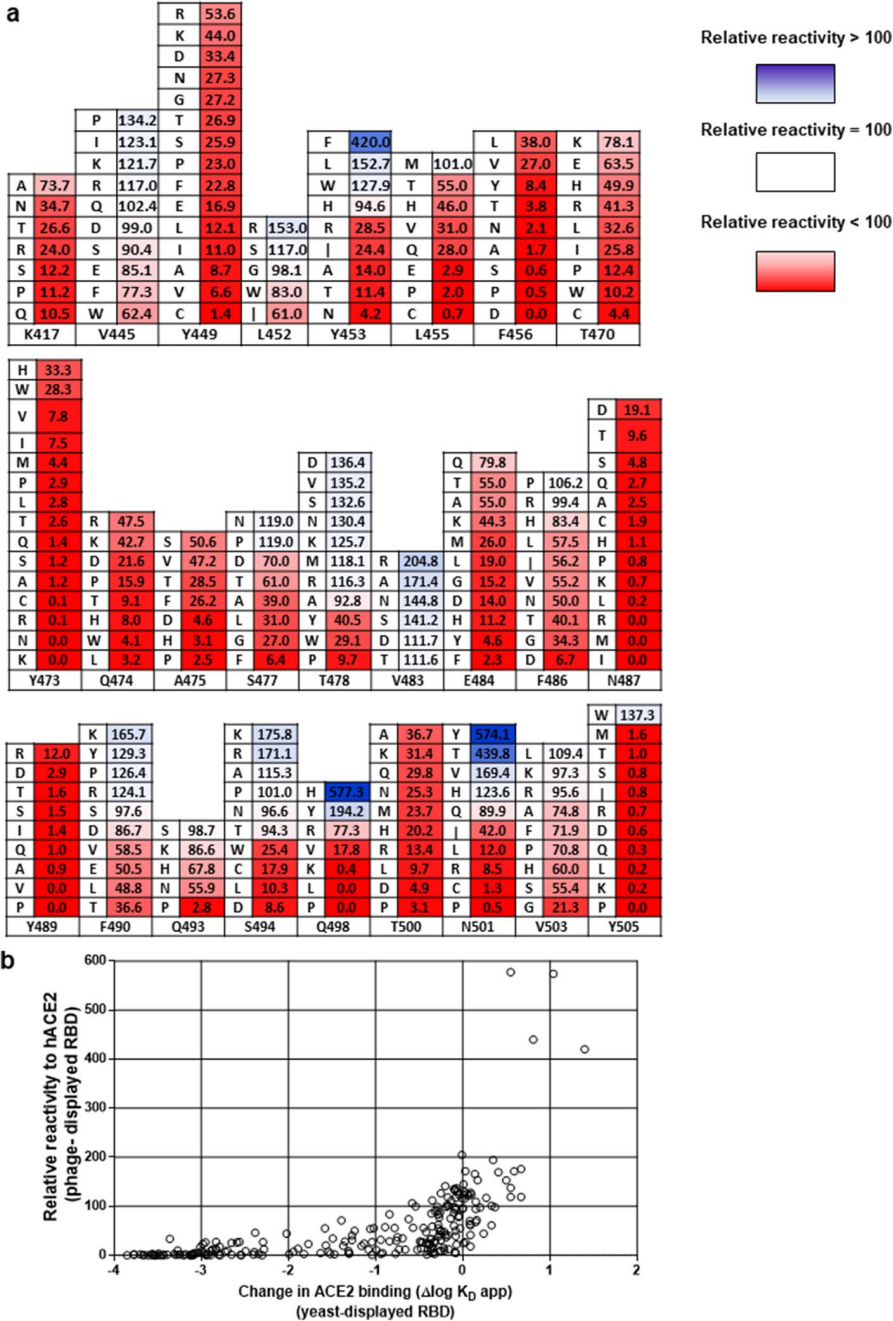
Mutational scanning of phage-displayed RBD. Individual randomization of 26 positions in Wuhan-Hu-1 RBD was carried out using Kunkel mutagenesis on the phagemid template with degenerate oligonucleotides introducing an NNK triplet. A total of 242 unique single-mutated sequences without other undesired changes were retrieved, and used to produce/purify phages, which were tested by ELISA on polyvinyl chloride microplates coated with a fusion protein comprising human ACE2 extracellular domain fused to human IgG1 Fc domain and with the anti-tag 9E10 mAb. Bound phages were detected with an anti-M13 PVIII antibody conjugated to horseradish peroxidase. Receptor reactivity of each clone was normalized according to its protein display level, assessed through recognition by 9E10. Relative reactivities were then calculated as the ratio between normalized reactivity of a given clone and that of the original Wuhan-Hu-1 phage-displayed RBD used as a reference. Replacements by different amino acids at each targeted position are shown (**a**). The numbers indicate relative reactivity value (%) of each variant. Color intensity is proportional to the magnitude of binding increases (blue) or decreases (red) in comparison with the reference RBD. Relative ACE2 reactivity values assessed with the current phage-based method were shown to correlate positively with the apparent K_D_ values of the same mutated RBD variants estimated using yeast display screening (**b**). Kendall’s Tau correlation coefficient was 0.6325.

A complex pattern of response to mutations was found (Fig. 4a). Most replacements at positions 417, 449, 455, 456, 470, 473, 474, 475, 484, 486, 487, 489, 493 and 500 reduced receptor binding to certain extent, indicating that these 14 residues contribute to the formation of the functional interface with human ACE2. Only the conservative mutation L455M, and the changes F486P/R/H and Q493S/K, were highly tolerated, keeping more than 80% of the original binding. While 77 mutations at these positions reduced binding to less than 20%, 53 changes resulted in partial reactivity (between 20 and 80%). Disruptive effects of mutations were particularly striking at positions 487 and 489, where all tested replacements caused an important binding loss, and several abolished ACE2 recognition, indicating an individually critical role for N487 and Y489. The situation for F456 and Y473 was similar, with multiple substitutions resulting in a drastic binding loss. The exceptions were F456L/V and Y473H/W, keeping hydrophobicity at position 456 and aromatic character at position 473, and retaining partial reactivity to ACE2. It is very difficult to segregate totally the effects of mutations on binding from their impact on protein folding and stability. Not all disruptive mutations necessarily target residues directly engaged in interactions with the receptor, some might contribute indirectly to the formation of the functional interface through effects on local folding and protein structure dynamics.

Some replacements at positions 445, 452, 453, 477, 478, 490, 494, 498, 501, 503 and 505 increased ACE2 binding, while several substitutions at the same locations affected recognition. The original residues at these positions thus contribute to the formation of the functional interface, but plasticity of the binding domain allows remodeling and optimization of the interaction. Y505 can be classified as a critical residue, being unique within this group. Although the replacement by a second aromatic aa (Trp) improved binding, all the other substitutions diminished reactivity down to negligible levels (below 2%). The remaining ten positions within this group exhibited a broad range of tolerance to mutations, including 34 interaction-enhancing replacements, 14 highly tolerated (80–100% reactivity), 25 partially tolerated (20–80%) and 16 highly disruptive (< 20%) substitutions. Residue V483 was unique among the group of targeted aa, because all the explored replacements led to ACE2 binding increases, despite drastic changes of side chain physical–chemical properties. The largest binding improvements among the whole set of mutations were associated to replacements Y453F, Q498H, N501Y and N501T.

There were differences between the dynamic ranges of our phage-based ACE2 binding assay and previous estimations of apparent K_D_ values derived from the statistical analysis of yeast display output^24^. While the scale of the phage assay (0–100%) determines that a severe loss in binding ability is visualized as a minimal signal, plotting the values of estimated K_D_ differences in a log scale still reflects differences between very weak binders (Fig. 4b). Nevertheless, there was a positive correlation between relative reactivity of phage-displayed mutated variants against human ACE2 and their apparent K_D_ values estimated using yeast display screening. A significant value (*p*˂0.05) was obtained for Kendall’s Tau correlation coefficient: 0.6325, validating the usefulness of phage display screening against an established reference method.

### Single mutations in RBD had different impact on inhibition of binding to ACE2 by human polyclonal antibodies

**Figure 5:**
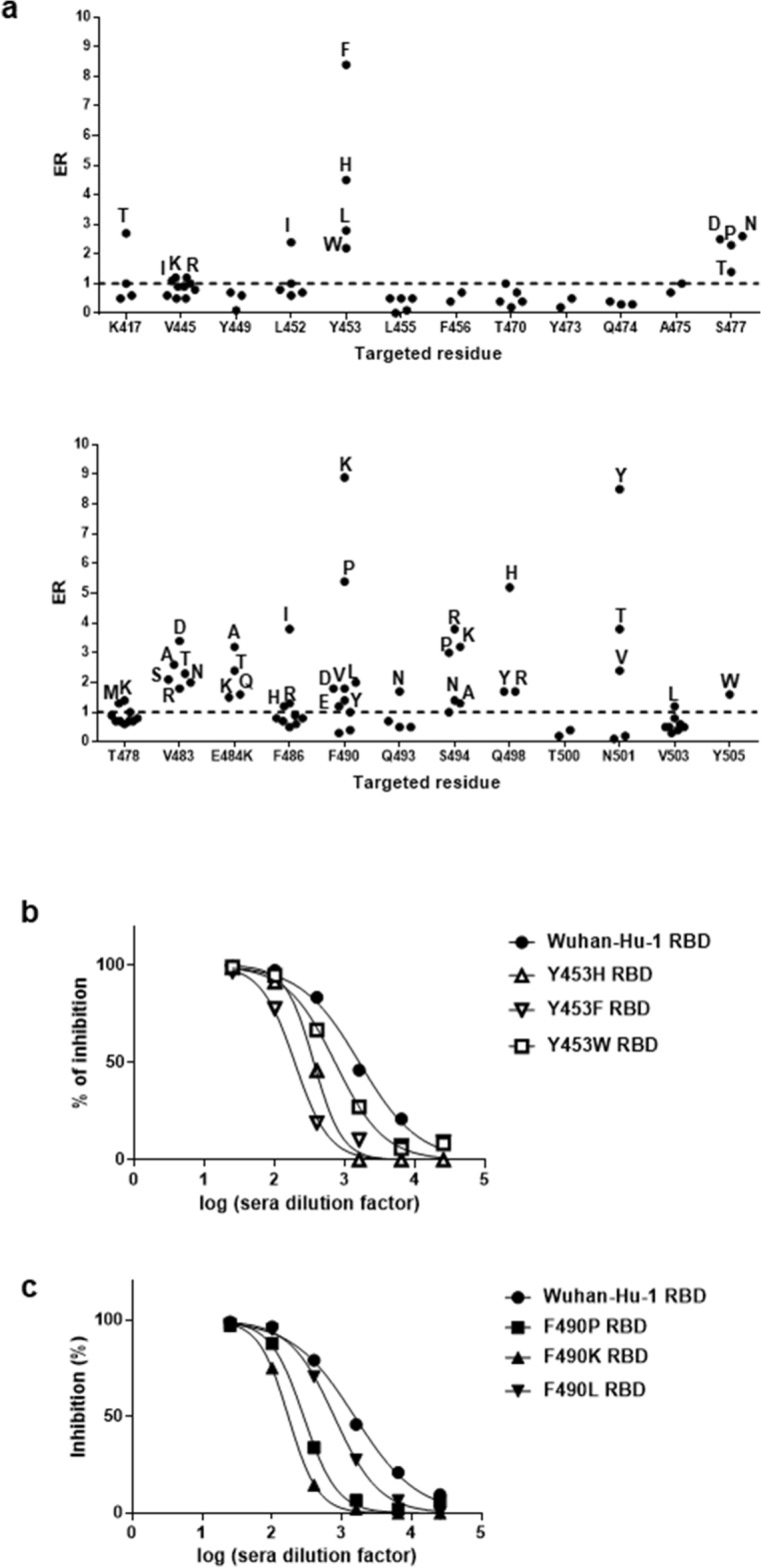
Effect of RBD mutations on inhibition of ACE2 binding by human sera. A pool of sera from 160 individuals who had received the heterologous vaccination scheme including two doses of SOBERANA 02 and one dose of SOBERANA Plus^9^ (without excluding individuals infected at any time during pandemic), was prepared. The capacity of this pool to inhibit the interaction between phage-displayed mutated RBD variants and ACE2 was assessed by ELISA. Sub-saturant amounts of normalized phage-displayed mutated RBDs were pre-incubated with serial dilutions of the pool of sera. Similarly diluted phage samples were used as reference (100% binding). Samples were incubated on polyvinyl chloride microplates coated with the recombinant fusion protein comprising the extracellular domain of human ACE2 fused to a human IgG1 Fc domain. Bound phages were detected with an anti-M13 PVIII antibody conjugated to horseradish peroxidase. Data were fitted to sigmoidal inhibition curves, and half-maximal inhibitory sera dilutions (ID50) were estimated. The ratios between the ID50 value determined for the original phage-displayed Wuhan-Hu-1 RBD and the ones calculated for every mutated RBD were calculated and plotted (**a**). An escape ratio (ER) value > 1 indicates a diminished ability of sera to inhibit binding of a given variant, while ER ≤ 1 corresponds to equal or higher sera inhibition capacity as compared to the original RBD. The dotted line represents the cut-off value (ER = 1). Replacing amino acids resulting in ER > 1 are identified with letters. Inhibition curves of selected phage-displayed mutated variants with high ER values are shown in **b** (replacements at position 453) and **c** (changes at position 490).

The occurrence of mutations in SARS-CoV-2 RBD has implications beyond their effects on ACE2 binding. Assessing the impact of RBD changes on antigenicity can be useful to understand and/or anticipate the complex interplay between viral evolution and immunized human populations. A pool of sera from 160 Cuban individuals who had received the heterologous vaccination scheme with two doses of SOBERANA 02 and one dose of SOBERANA Plus^9^ (some of them infected with SARS-CoV-2 at any time during pandemic) was chosen as the probe to study the antigenicity of mutated RBD variants. The ratio between sera inhibitory capacity (ID50) on ACE2 binding of the original non-mutated phage-displayed RBD and its counterpart containing a given mutation was called ER (escape ratio), and used to measure the potential of such mutated RBD to avoid neutralization by pre-existing antibodies (Suppl. Table S1). A variant with an ER > 1 is less inhibited than the original RBD, whereas a variant with an ER < 1 is more susceptible to inhibition (Fig. 5a). Only those mutated variants showing total or partial ACE2 reactivity conservation, as well as those exhibiting binding enhancement, were analyzed.

Antibodies contained in the pool of immune sera prevented binding of all the single-mutated RBD variants tested, reinforcing the idea that polyclonal responses to vaccination/infection simultaneously target multiple neutralizing epitopes. Sera inhibitory capacity varied depending on the mutation. Most mutations (68) resulted in equal or higher inhibition of the corresponding RBD variants as compared to the original domain. The remaining replacements (49) affected sera inhibitory capacity, albeit at different extents. None of the tested changes at positions 449, 455, 456, 470, 473, 474, 475, and 500 decreased sera-mediated inhibition. Positions 445, 478, 493 and 503 exhibited a mixed pattern of response to mutations, ranging from enhanced or conserved to slightly decreased inhibition. A moderate inhibition loss was observed when Y505 was replaced by Trp.

All tested changes at positions 453, 477, 483, 484 and 498 diminished inhibition by immune sera. The same happened with all but one replacement at position 494. These effects were not solely due to the barrier imposed by an enhanced ACE2 binding, as the reduction in inhibitory activity was observed for variants having either increased or partially decreased binding ability. All mutations targeting residue 484 diminished both ACE2 binding and inhibitory power of sera, highlighting the relevance of pure antigenicity loss. Some replacements at positions 417, 452, 486, 490 and 501 resulted in escape from pre-existing antibodies, despite conserved binding inhibition of RBD variants with other changes at the same locations. Divergence between inhibition and increased ACE2 binding was observed again. An interesting exception was the set of mutations targeting residue 501, as their ranking of inhibition loss reproduces the one of increased ACE2 binding, pointing to enhanced affinity as an important cause of escape from antibody-mediated inhibition due to changes at this position. The four strongest ACE2 binding enhancing mutations (Y453F, Q498H, N501Y, N501T) were among the main changes reducing inhibition. Y453H, V483D, E484A, F486I, F490K, F490P, S494K, S494R and S494P also reduced antibody-mediated inhibition by more than threefold.

Remarkably, none of the tested RBD mutations caused a total loss of sera-mediated ACE2 binding inhibition. Even those variants with the highest ER ratios were inhibited, albeit at lower sera dilutions, as illustrated in Fig. 5b, c. This result supported the notion of strong humoral responses against a single RBD vaccine antigen being able to cross-neutralize cell entry of emerging viral variants, at least to certain extent.

### In silico exploration of the structure–function relationships highlighted by phage display screening

**Figure 6:**
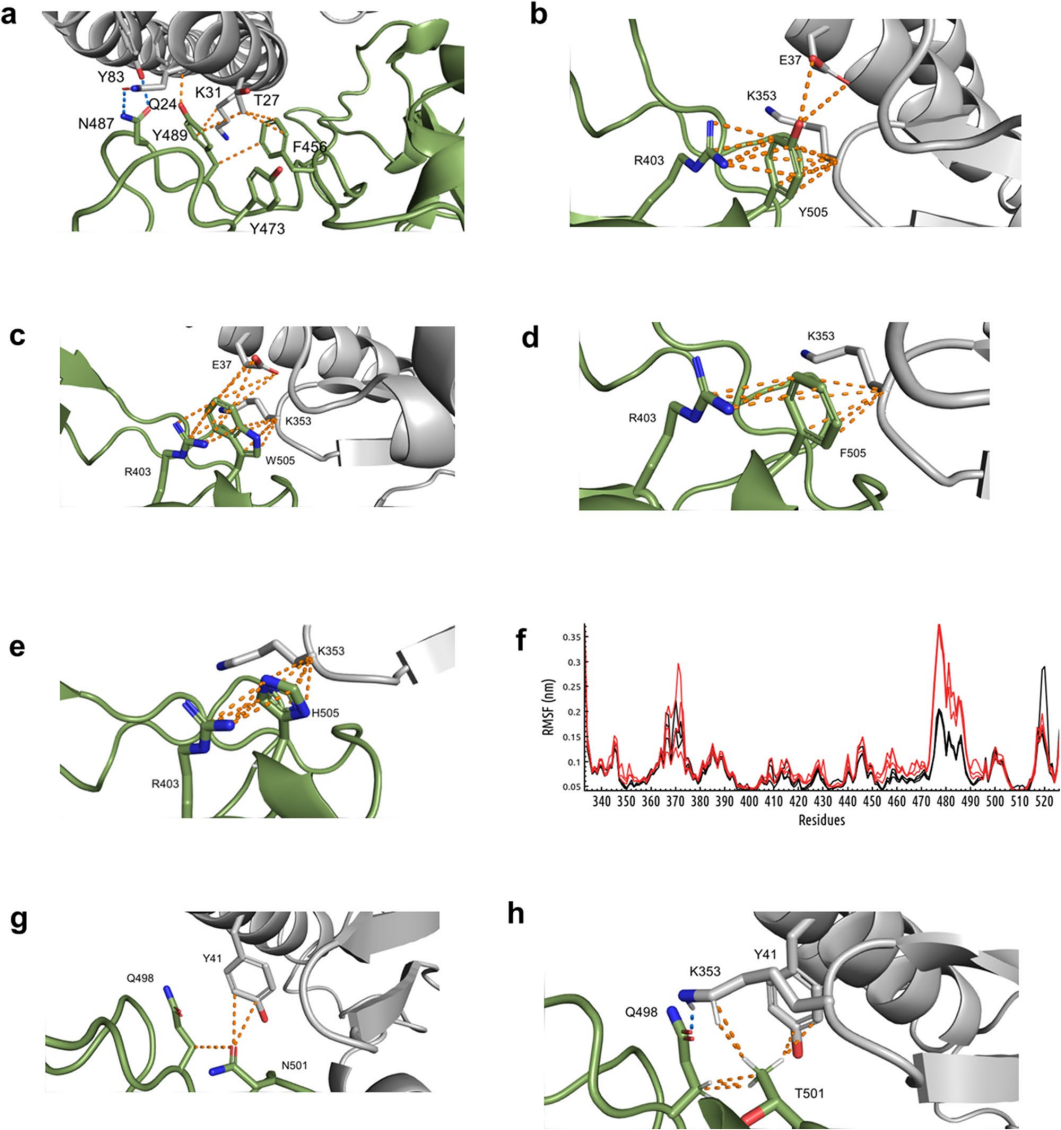
Structural bases of the effects of RBD mutations on ACE2 binding. The structure of the RBD/hACE2 complex was extracted from the Protein Data Bank (PDB ID: 6M0J). Molecular modeling of mutated RBD variants and relaxation steps were performed using Rosetta. Visualization and analysis of the structures were performed with Pymol (**a–e**, **g**, **h**). The interface between human ACE2 (grey) and SARS-CoV-2 RBD (green) is shown. Both molecules are represented as cartoons. Side chains of the residues relevant for the analysis are shown with sticks, colored by element and labelled. Polar contacts appear as yellow dotted lines, and blue dotted lines indicate hydrogen bonds. Wuhan-Hu-1 RBD and its Y473A mutated variant (extracted from the complexes) were studied by molecular dynamics simulations during 50ns using Gromacs 2022.3 and the CHARMM-36 forcefield. RMSF plots (**f**) show fluctuation along the primary sequence of three replicates of each protein. Overlapping RMSF curves corresponding to the original Wuhan-Hu-1 RBD appear in black, while Y473A plots are represented in red.

The contribution of some of the few residues in RBD identified as critical in the above-described mutagenesis studies is illustrated in Fig. 6a. N487 side chain forms two hydrogen bonds with Y83 and Q24 in ACE2. Y489 and F456 are engaged in a network of multiple non-bonded contacts between them and with T27 and K31 of ACE2, which could stabilize the interaction. Y505 (Fig. 6b) establishes multiple non-bonded contacts with ACE2 and is also engaged in a cation-pi interaction with R403 within RBD itself. The latter could contribute to RBD structure stabilization. A second aromatic residue (Trp) was able to fulfill similar structural requirements (Fig. 6c), which is consistent with the moderate binding enhancement observed experimentally upon Y505W replacement. Other aromatic aa (Phe and His) also kept the interacting capacity (Fig. 6d, e). Y473, although not directly involved in inter-molecular contacts (Fig. 6a), was shown to restrict flexibility of the RBD segment 450–490, potentially favoring the accommodation of both interacting partners. Its replacement by Ala resulted in higher mobility of this segment during molecular dynamics simulations using free RBD extracted from the complex (Fig. 6f).

The structural bases of large increases of RBD binding ability to ACE2 associated to mutations at positions 453, 498 and 501 have been explored previously^21,39–42^. In the case of N501T, our modeling studies indicated that T501 keeps the contacts with Y41 from ACE2 and Q498 within the RBD itself established by N501 (Fig. 6g), but also interacts with ACE2 K353 (Fig. 6h). Besides the possible impact of this new contact on binding, it accommodates K353 side chain in close proximity to RBD Q498, resulting in the formation of a hydrogen bond between them (Fig. 6h), which could strength the interaction further.

**Figure 7:**
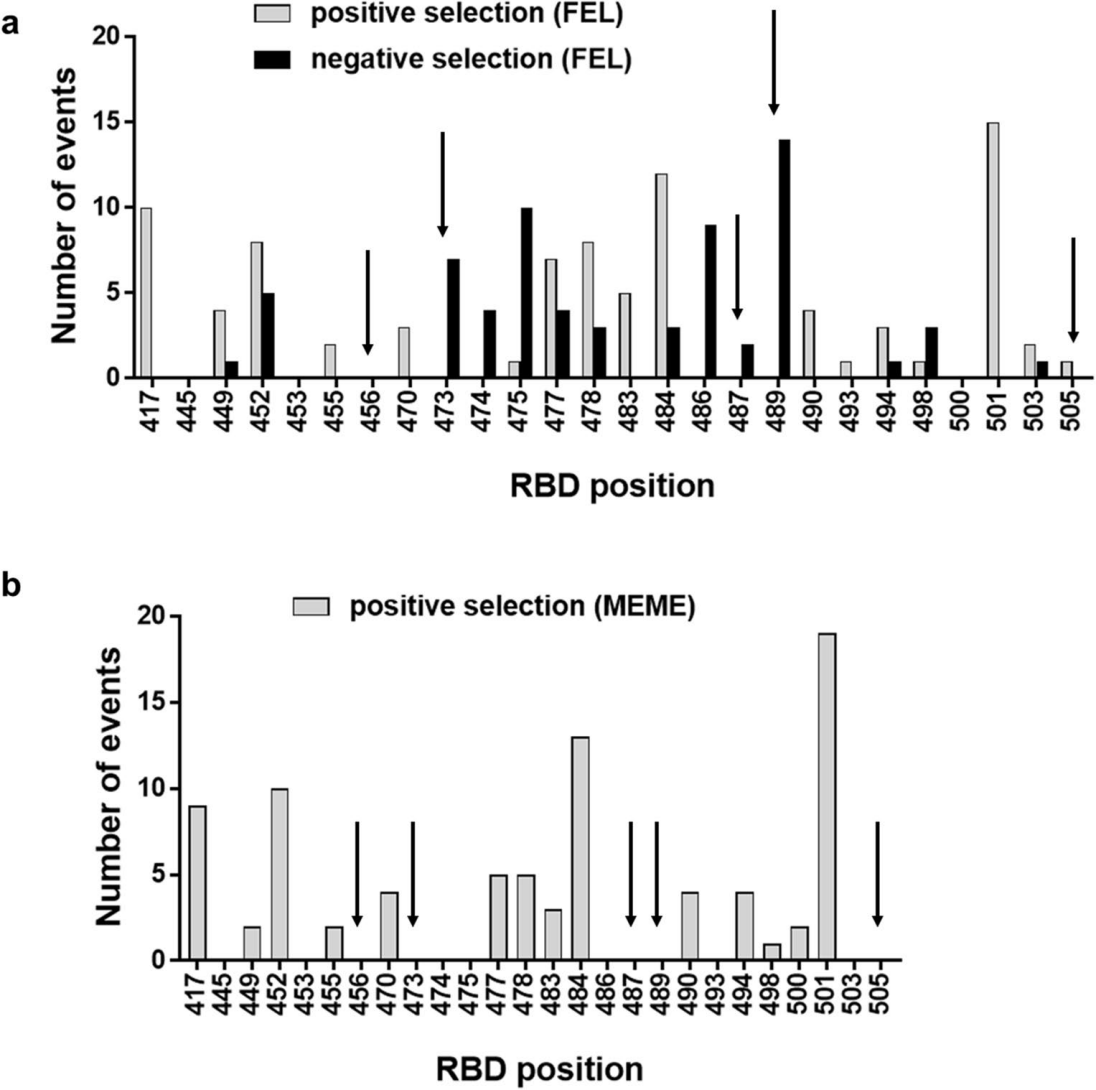
Selection events associated to replacements at selected positions along the RBD sequence. A dataset comprising SARS-CoV-2 genomic sequences from GISAID and processed by a SARS-CoV-2 analysis pipeline (https://github.com/veg/SARS-CoV-2/) was taken from https://github.com/spond/SARS-CoV-2-variation (DOI: https://zenodo.org/badge/latestdoi/378638985). After the alignment to the reference genes, and the process of compressing and filtering the data, all sequences were partitioned into 90-days intervals by sampling dates, maximum likelihood phylogenies were reconstructed for each time interval, and gene-by-gene distances were estimated to compute diversity and divergence using TN93 distance. Several HyPhy dN/dS-based selection analyses were run on each gene. The analyses, aimed at identifying sites that may be experiencing selection-driven diversification and restricted to internal branches of the tree, included fixed events likelihood (FEL, **a**) for pervasive selection, and mixed effects model of evolution (MEME, **b**) for episodic selection. The above-described dataset was searched to look for such sites of positive selection events (MEME/FEL *p* values < 0.05) and also negative selection in the case of FEL, among RBD positions targeted in the phage-based mutational scanning experiments. Search was limited to the period between December 2019 and October 2021. Each event represents the occurrence of a significant signal of selection within the preceding 3-months period.

Establishing a direct relationship between the effects of RBD mutations on ACE2 binding ability and their appearance during pandemic is not possible, due to the complex interplay between sequence changes within the RBD and in the whole spike protein, other genome modifications, and selective pressures acting at multiple levels of viral infection. However, a close inspection of collections of viral sequences revealed a comprehensive landscape of positive and negative selection events associated to modifications at certain RBD positions during viral spreading. The analysis was restricted to RBD residues previously targeted by mutagenesis and to the period December 2019- October 2021, since subsequent emergence of the Omicron family (Suppl. Fig. S6), containing multiple RBD replacements, would have complicated the analysis even further. Modifications at the five positions identified as critical for RBD binding in our study (456, 473, 487, 489 and 505) were not positively selected in general. This was shown by using both fixed events likelihood analysis (FEL, Fig. 7a) and mixed effects model of evolution (MEME, Fig. 7b) to identify sites of adaptive evolution. Diversification at some of them resulted in negative selection events (Fig. 7a). The only positively selected diversifying event at position 505 involved the replacement by a second residue having an aromatic ring (His), which was previously shown to be the crucial moiety at this location. The above-described findings highlighted the biological relevance of critical RBD features identified through phage display screening.

## Discussion

Phage-displayed Spike protein fragments had been previously used for epitope mapping. In these works, multiple random fragments were selected from SARS-CoV-2 genomic libraries by antibodies from vaccinated animals and from infected humans^43,44^. The goal was the identification of immunogenic epitopes present in overlapping segments enriched after panning. The current work differs from these examples, since a single fragment from SARS-CoV-2 Spike protein, comprising the whole RBD, was designed a priori, in order to reproduce domain native folding. Residues critical for the interactions with either the receptor or antibodies were not dissected by further segmentation, but through the introduction of single/multiple aa replacements in the fully folded domain.

Despite RBD has often been defined as the sequence between residues 319 and 541 of S protein, the minimal RBD sequence (aa 331–524)^11^ plus short extensions at both N- and C-termini (aa 328–533) was chosen for display. This segment includes the compact folding unit corresponding to RBD and the eight Cys residues that form disulfide bonds keeping its architecture. The unpaired Cys 538, which could cause non-natural disulfide bond formation, was excluded. One of the major concerns when displaying glycoproteins is the lack of glycosylation in *E.* *coli*, where phages are assembled. Display of functional and antigenic RBD, shown by its ability to bind ACE2, anti-RBD mAbs and anti-viral polyclonal antisera, is in agreement with previous reports about the conservation of the structure, antigenicity and biological activity of recombinant RBD produced in diverse hosts with different glycan composition^24,45^ and even aglycosylated^27^. Although N-linked glycans of RBD are not essential to keep its interactions, the presence of carbohydrate moieties at positions 331 and 343 has been postulated to improve protein stability^24^. However, lack of glycosylation did not preclude successful display of RBD on phages at levels good enough for analytical purposes.

Validation of phage-displayed RBD as a model antigen mimicking the interactions of the natural viral domain had immediate application. To our knowledge, the first reported use of this strategy was the assessment in our laboratory of cross-inhibition of binding of VOC RBD to ACE2 by antibodies raised by Cuban subunit vaccines^9,46^. This was accomplished before cell-based viral neutralization assays were locally available. Cross-inhibition shown with the phage-based ELISA, as well as the moderate loss of inhibitory power against mutated RBD variants (particularly Beta RBD) were subsequently confirmed by classical neutralization assays^47^. Phage display was subsequently used by other authors to study humoral responses to mutated RBD variants, but their approach differed from the current one in display multivalency (RBD proteins were fused to all copies of PIII due to gene insertion into the phage genome) and in the assay format (phage display mediated immuno-multiplex quantitative PCR)^48^.

An additional application of our methodology was mutational scanning of Wuhan-Hu-1 RBD. Phage-based functional exploration of RBD/ACE2 binding interface differs from previous yeast display approaches^24^. First, our work provided a straightforward solution for the direct measurement of the effects of hundreds of individual RBD mutations on ACE2 binding. Monovalent display on phage PIII and normalization based on display levels minimize the influence of avidity effects and expression differences in the results. Monovalency was achieved on statistical bases, through the use of a phagemid-based system where native PIII from helper phage effectively competes with the limited amounts of PIII fused to the heterologous proteins for incorporation into viral particles. Availability of the fusion proteins is kept at minimum because of the low leaky expression levels under the control of *Lac* promoter in the absence of any inductor compound (just by removing glucose from the media), and the insertion of an amber stop codon between RBD-*c-myc* and PIII sequences, which is partially suppressed in TG1 strain, often resulting in incomplete protein synthesis. The simplicity of the current approach contrasts with the statistical inference of apparent K_D_ values for yeast-displayed mutated variants based on their frequency among populations sorted with decreasing ACE2 concentrations^24^. Since many variants contained multiple mutations (2.7 on average), the use of global epistasis models was required to determine the effects of individual mutations. A second difference was the limited coverage of the current study, restricted to a partial mutational scanning of only 26 positions within phage-displayed RBD through manual processing. Deep mutational scanning of large yeast libraries, comprising 10^5^ members, through flow cytometry and massive DNA sequencing provided a complete functional map of RBD^24^. Sequence space coverage was thus comprehensive, as 3804 of 3819 possible RBD mutations were represented in the library. The fact that measurements done with both display platforms showed a positive correlation, and led to similar conclusions (see below) provided a cross-validation of the results. Future approaches might take advantage of the automation amenability and high throughput screening potential of phage display libraries, which typically reach sizes above 10^9^ molecules. Combining these tools with next generation DNA sequencing could represent a step forward in the exploration of RBD diversity.

The results of phage-based screening are in good agreement with previous mutagenesis studies, and with structural and viral molecular evolution analyses. Remarkable plasticity of RBD/ACE2 interface, which is totally or partially tolerant to many individual replacements, is consistent with the initial estimation that a substantial mutational space is compatible with enough ACE2 affinity to maintain human infectivity^24^, and with the fast accumulation of RBD mutations during COVID-19 pandemic. The few individually critical positions identified in our study (F456, Y473, N487, Y489, Y505) also emerged as such from yeast-based screening^24^. Selective tolerance to replacements involving hydrophobic and aromatic residues at positions 456 and Y473 respectively, and moderate binding enhancement associated to replacement Y505W, were observed in both systems^24^. The four major RBD-ACE2 interaction-enhancing mutations detected in our study corresponded to changes already observed in nature and/or identified in other experimental settings^24^.

The replacement Y453F was detected in virus isolates from an outbreak in mink farms in Denmark^49^, and shown to increase binding affinity to both mink and human ACE2^39,40^. N501Y has been extensively characterized as an ACE2 affinity-increasing replacement^21^ and was the only RBD mutation fixed in the first SARS-CoV-2 VOC known as Alpha^16^. It also appeared in Beta^17^, Gamma^18^ and in the expanding Omicron family^20^ of VOC, playing a dominant role in viral evolution. Its emergence was reproduced in yeast-displayed RBD directed evolution experiments^50^. N501T also enhanced ACE2 binding despite the divergent chemical nature of the replacing aa. This result matches with previous experimental^40^ and in silico analysis^41^ of this mutated variant. N501T appeared eventually in nature during viral transfers between minks and humans^51^, but was not fixed due to the strong predominance of N501Y. Q498H, the fourth replacement causing a large binding increase, deserves further attention. Despite the clear evidences of the intrinsic ACE2 binding enhancement effect of this change, and its emergence during directed evolution simulations in vitro using cell surface display^42^, the presence of His at this position is not compatible with N501Y^42^. The simultaneous presence of N501Y and Q498R was observed instead in both directed evolution experiments^42,50^ and in the Omicron family of variants which expanded all over the world^20^, presumably due to synergistic effects of both replacements^52^.

Two different mechanisms could mediate RBD escape from neutralization by sera of vaccinated individuals. The first is the stronger ACE2 binding ability of RBD variants, which would impose a barrier for antibodies to compete with the receptor. Inhibition escape was indeed shown here for the four above-described RBD variants containing binding-enhancement mutations. This effect on competition would be seen even if the mutations do not target the site recognized by the antibodies. The second mechanism of escape would be directly related to the lack of recognition by neutralizing antibodies when replacements disrupt RBD target epitopes. This could happen even when a given replacement results in partial loss of ACE2 binding capacity. Multiple mutations at position 484, identified in the current study, illustrate pure antigenicity loss, not linked to ACE2 affinity-enhancement, as the cause of escape from inhibitory activity of sera.

Some previous studies using yeast-displayed RBD relied on the mere lack of recognition by monoclonal/polyclonal antibodies, followed by the assessment of the impact of selected mutations on neutralization^25,26^. Other authors have simulated viral escape in a more realistic competitive system, assessing yeast-displayed RBD binding to ACE2 in the presence of neutralizing antibodies^27^, which is similar to our own format. The emergence of escape variants has also been modelled in biological scenarios, where the whole host-virus interplay is recapitulated with chimeric viruses whose infectivity depends upon the recombinant expression of SARS-CoV-2 spike protein^37,53^. Beyond the technical differences between diverse systems, it is important to note that the use of pooled sera restricted the scope of the current work to the identification of mutations having the largest and/or most prevalent effects within the population under study. This allowed a global view of the interplay between RBD variability and the immune system of a subset of Cuban vaccinated individuals, and underscored critical mutational hotspots that could reduce protection at the population level. Automation and high throughput screening of multiple individual sera could reveal specific antibody signatures, known to differ among human subjects^26^.

Despite the above-described methodological singularities, and the unique features of the Cuban population and vaccination protocols, our results fit into the global landscape of mutational escape from antibodies. Position 484 had been already highlighted as a prominent site of appearance of escape mutations which preclude recognition and/or neutralization by mAbs and antisera from both convalescent patients and vaccinated individuals^25–27,53^. Diverse changes at this position have been indeed fixed in VOC RBD^17,18,20^_._ Some replacements at the neighbor position 483 have been shown to confer decreased sensitivity to mAbs or convalescent sera^37^. Most RBD positions at which escape mutations were detected in the current study (417, 445, 477, 478, 483, 484, 486, 490, 493, 494, 498, 501, 503 and 505) had already been reported as sites where one or more replacements affect recognition and/or neutralization by human antibodies to certain extent^26,27,37,53^.

Taken as a whole, our findings point to the coexistence of multiple potential sites of partial immune escape within RBD with a largely conserved ability of polyclonal antisera to inhibit binding of individually mutated variants to ACE2 receptor. It will be important to watch the appearance of some specific RBD sequence changes conferring the largest resistance to antibody-mediated receptor binding inhibition, as well as the emergence of new combinations of mutations which could cause additive or synergistic escape effects. However, the general scenario is consistent with the effectiveness of sera from children who have received two doses of SOBERANA 02 and one dose of SOBERANA Plus to neutralize SARS-CoV-2 VOC, including Omicron^54^.

The problems to obtain biologically active Omicron BA.1 RBD illustrated how mutations affect protein stability and folding, precluding production in recombinant hosts. Expression constraints within yeast-displayed RBD mutational space had been previously explored^24^. The only individual aa replacement present in the BA.1 variant that affected expression in that study was S375F. The behavior of BA.1 RBD could thus be due to S375F and/or to combinatorial effects arising from the accumulation of mutations. Our results raised concerns about possible difficulties to obtain recombinant Omicron RBD antigens for vaccination or other purposes. Both BA.1 and BA.2 have indeed been shown to have reduced yeast surface expression levels as compared to Wuhan-Hu-1 RBD^55^. Stabilizing mutations in the Omicron RBD core (positions 358, 363, 365, 392) and around some of the Omicron substitutions (positions 369, 374, 376) have been identified using yeast display^55^. Phage display is exquisitely sensitive to the intrinsic instability and aggregation propensity of the proteins^33^, and has led to the discovery of variants exhibiting improved secretion and folding not revealed through other platforms^34,56^. Our failure to display BA.1 RBD on phages can thus be seen as an opportunity to decipher the molecular bases of its biophysical liabilities and to further optimize its developability profile. Directed evolution of SARS-CoV-2 RBD variants to obtain stable and well-folded recombinant proteins with good manufacturability profiles is thus another potential application of our phage-based approach.

Exploration of the mutational space surrounding SARS-CoV-2 phage-displayed RBD has been limited to known combinations of replacements present in VOC and a small set of single substitutions within RBM. The construction of large libraries of RBD variants using diversification strategies like soft-randomization or error-prone PCR, together with suitable enrichment procedures, could simulate in vitro the natural evolution of RBD under selection pressures related to receptor binding and/or escape from neutralizing antibodies. Since viral variants with multiple simultaneous mutations are already dominating SARS-CoV-2 evolutionary landscape, expanding our ability to reproduce such a massive diversification in the phage-based platform will be crucial to reveal complex epistatic effects on receptor binding and antigenicity. This goal should be technically achievable, given the previous experience in creating large phage-displayed combinatorial libraries of other molecules^28^. This strategy could recapitulate what already happened during pandemic, but also predict possible pathways of future viral evolution, while infection is still around. Since coronaviruses had been considered a serious and long-term health threat, even before COVID-19^57^, functional mapping and in vitro evolution of their phage-displayed receptor binding domains are attractive goals. Despite the diversity of RBD structures with binding capacity to different cell receptors on a wide range of hosts, the shared basic RBD fold among the betacoronaviruses (a single-layer β-sheet core with a loop comprising RBM) and the similar topology of more divergent receptor binding domains in other coronaviruses^57^, point to the feasibility of expanding the current experience beyond SARS-CoV-2.

## Methods

### Phage display of SARS-CoV-2 RBD and its mutated variants

The genes coding for SARS-CoV-2 Wuhan-Hu-1 (aa 328–533) RBD and its Omicron BA.1 counterpart were synthesized by Eurofins™ and cloned into the pCSM phagemid vector^31^, fused to the phage protein III gene, using flanking ApaLI and NotI restriction sites (Fig. 1). The remaining mutated VOC RBD genes (Alpha, Beta and Delta) were constructed by site-directed mutagenesis of the original Wuhan-Hu-1 RBD gene using oligonucleotides (CIGB, Cuba) containing the desired changes. The sequences of all cloned genes were confirmed by Microsynth-Seqlab, Germany. TG1 *E.* *coli* cells (K12_(*lac-pro*), *sup*E, *thi*, *hsd*D5/F’ *tra*D36, *pro*A^+^B^+^, *lac*I^q^, *lac*Z_M15) were transformed with each genetic construct, and phage particles were rescued with M13KO7 helper phage at a 50 mL scale and purified by precipitation with polyethylene glycol using established procedures^31^. Relative protein display levels were determined by ELISA on polyvinyl chloride microtitration plates coated with the anti-*c-myc* tag 9E10 antibody as described^31^. A standard curve of diluted phages displaying Wuhan-Hu-1 RBD was used as reference, assuming that the display level of this phage sample was 100 arbitrary display units/mL.

### Phage ELISA to assess RBD antigenicity and biological activity

Polyvinyl chloride microtitration plates were coated overnight at 4 °C with anti-*c-myc* tag 9E10, anti-RBD mAbs (CIGB Sancti Spiritus, Cuba) or the recombinant protein comprising the ECD of human ACE2 fused to human IgG1 Fc (hACE2-hFc, produced at the Chimeric Proteins laboratory of the Center of Molecular Immunology, CIM) diluted in PBS at 5 μg/mL. The plate was blocked with phosphate-buffered saline (PBS) containing 4% (w:v) skim milk powder (M-PBS) during 30 min at room temperature (RT). Phage samples were appropriately diluted in M-PBS and incubated during 1 h at RT on coated/blocked plates. In order to compare the antigenicity and biological activity of different phage-displayed RBD mutated variants, equivalent concentrations of them (determined by ELISA on 9E10-coated plates as described in the previous section) were used. Plates were washed with PBS containing 0.1% (v:v) Tween 20 (PBS-T), and bound phages were detected with an anti-M13 antibody targeting PVIII conjugated to horseradish peroxidase (HRP) properly diluted in M-PBS. After 1 h of incubation, plates were washed as described, and peroxidase substrate solution (ortho-phenylenediamine at 0.5 mg/mL and 0.015% (v:v) hydrogen peroxide in 0.1 mol/l citrate–phosphate buffer, pH 5) was added. After 15 min the reaction was stopped with 10% (v:v) sulfuric acid. The absorbance at 490 nm was measured in a microplate reader.

### Mutational scanning of selected positions within phage-displayed RBD

Randomization of individual positions within RBD gene was performed through multiple separate Kunkel mutagenesis^35^ reactions on the single-stranded phagemid template containing Wuhan-Hu-1 RBD gene, using mutagenic degenerate oligonucleotides which introduce an NNK codon at each targeted site^58^. TG1 *E.* *coli* cells were transformed with mutagenesis products. Phages displaying mutated products were rescued from isolated colonies at a 96-well plate scale using M13KO7 helper phage^58^. Phage-containing supernatants (three-fold diluted in M-PBS) were tested by ELISA on plates coated with either 9E10 mAb or hACE2-hFc fusion protein, as described in the previous section. Normalized reactivity against ACE2 was calculated as the ratio between the absorbances obtained for a given clone on hACE2-hFc and on 9E10 mAb-coated wells. Relative reactivity for each clone derived from mutagenesis was determined as the ratio between its normalized reactivity and the one of control phages displaying Wuhan-Hu-1 RBD, which were produced in parallel in the same 96-well plate.

The above-described screening allowed a gross classification of mutated variants in three groups: those showing decreased, conserved or increased ACE2 relative reactivity in comparison to the original phage-displayed Wuhan-Hu-1 RBD. Variants with different levels of relative reactivity were thus selected for sequencing. XL-1 Blue cells (*rec*A1 *end*A1 *gyr*A96 *thi*-1 *hsd*R17 *sup*E44 *rel*A1 *lac* F´ *pro*AB l*ac*IqZ_M15 Tn10 Tet^r^) were infected with the corresponding phage-containing supernatants, and used to purify phagemid DNA with the QIAprep Spin minikit (Qiagen, Germany), following manufacturer's instructions. The RBD genes inserted in each plasmid were sequenced by Microsynth-Seqlab, Germany. Representative clones having unique aa replacements at each targeted position without undesired stop codons or other changes along the rest of the sequence were selected for further characterization. After TG1 *E.* *coli* transformation with these genetic constructs, phages were rescued at a 50 mL scale and purified as described above. Phage preparations were normalized considering their display levels (assessed by ELISA on 9E10-coated plates)^31^. Their ability to bind ACE2 was subsequently determined on microtitration plates coated with hACE2-hFc, and relative reactivity (%) was calculated taking the one of phage-displayed Wuhan-Hu-1 RBD as the reference (100%).

### Competition ELISA assays to assess the inhibition of binding of phage-displayed RBD variants to ACE2 by antibodies or other molecules

The assay format resembled that of the already described phage ELISA to assess biological activity of phage-displayed RBD. Purified phages displaying Wuhan-Hu-1 RBD (or a given RBD mutated variant) were diluted in M-PBS at sub-saturating concentrations (previously determined by the analysis of binding saturation curves on hACE2-hFc-coated polyvinyl chloride microtitration plates). Diluted phages were incubated during 1 h at RT in the presence or absence of different concentrations of the competitor molecule(s). After pre-incubation, samples were added to ACE-2-coated/blocked plates and the immunoenzymatic assay was continued as described above. Different kinds of competitors were used: recombinant versions of RBD and ACE2, anti-RBD mAbs and polyclonal antisera from infected and/or vaccinated humans. In the case of sera, after logarithmic transformation of dilutions, data were fitted to a non-linear curve (log(inhibitor) vs normalized response with variable slope) to determine the ID50 values (dilution that produces 50% inhibition of binding). Maximal phage binding (100%) was assessed in the absence of any competitor.

### Production of His-tagged RBD-derived proteins by transient transfection of HEK293T cells adapted to grow in suspension

The genes coding for all the RBD variants were amplified by polymerase chain reaction (PCR), and cloned into the pCMX-His expression vector, which contains the gene coding for a C-terminal hexahistidine tag, using BssHII and NotI restriction sites. The correct insertion of these sequences was confirmed by Mycrosynth-Seqlab, Germany. HEK-293 T cells adapted to grow in suspension were grown in Freestyle F-17 medium (ThermoFisher Scientific, USA) and transiently transfected with these genetic constructs using linear polyethyleneimine as transfecting agent following established procedures^59^. Cells were fed 48 h post-transfection, with fresh media and 20% (w:v) tryptone. One week after transfection the cell culture supernatants were collected and the 6xHis-tagged proteins were purified by immobilized metal affinity chromatography using a Ni–NTA matrix.

### ELISA to assess biological activity of His-tagged RBD recombinant proteins

Biological activity of the purified His-tagged RBD proteins (see the previous section) was assessed by ELISA on polyvinyl chloride microtitration plates coated overnight at 4 °C with hACE2-hFc (at 5 μg/mL in PBS) and subsequently blocked with M-PBS during 30 min at RT. His-tagged proteins were serially diluted in M-PBS (from 2 µg/mL  to 16 ng/mL) and incubated on coated/blocked plates 1 h at RT. After washing with PBS-T, an anti-His-tag antibody conjugated with HRP (properly diluted in M-PBS) was added and incubated 1h at RT. Plates were washed and peroxidase substrate solution (see above) was added. After 15 min the reaction was stopped with 10% (v:v) sulfuric acid. The absorbance at 490 nm was measured in a microplate reader.

### Generation of stable clones producing mouse Fc-fused versions of RBD

The genes coding for Wuhan-Hu-1 and Omicron BA.1 RBD (synthesized by Eurofins™) were cloned into the intermediate vector pCMX/mFc using the restriction sites BssHII/NotI. The whole cassettes of expression of these plasmids, including CMV promoter and the genes coding for a mouse IgG heavy chain signal peptide, the RBD of interest (aa 328–533) and a mouse IgG2a Fc region, were amplified by PCR and cloned into the lentiviral vector pL6WBlast (CIGB, Cuba) through XhoI and EcoRV restriction sites. The sequence of the inserted DNA fragments was confirmed by Microsynth-Seqlab, Germany. Lentiviral particles were assembled by adherent HEK-293 T cells co-transfected with each of these genetic constructs together with pLPI, pLPII and pLP/VSV-G auxiliary plasmids (Invitrogen, USA), and subsequently purified from supernatant through precipitation with polyethylene glycol. Viral particles were titrated by ELISA with DAVIH Ag p24 ELISA kit (LISIDA, Cuba). HEK-293 cells were grown in individual wells of 96-well cell culture plates containing DMEMF12 medium (Life Technologies, USA) supplemented with 5% (v:v) heat-inactivated fetal bovine serum, and infected three times every 24 h with lentiviral particles at a multiplicity of infection of 800. Transduced cells were diluted and expanded to 96-well plates in the presence of the selection drug blasticidin (2 µg/mL). Secreted fusion proteins in the supernatants were detected by ELISA on polyvinyl chloride microtitration plates coated with an anti-mouse IgG antibody. Bound fusion proteins were detected by an anti-mouse IgG antibody conjugated to horseradish peroxidase. Oligoclonal cell populations secreting the highest levels of each fusion protein were cloned by limiting dilution, and clones were screened using the same ELISA. The best producer clones were expanded, adapted to grow in suspension in the proprietary medium MB06 (serum-free), and used to produce the fusion proteins, which were purified through protein A affinity chromatography. Purified proteins were analysed by size exclusion chromatography in a TSKgelG3000SWXL column to assess their aggregation status.

### ELISA to assess biological activity of Fc-fused RBD recombinant proteins

Biological activity of the purified mouse Fc-fused RBD proteins (see the previous section) was assessed by ELISA on polyvinyl chloride microtitration plates coated overnight at 4 °C with hACE2-hFc (at 5 μg/mL in PBS) and subsequently blocked with M-PBS during 30 min at RT. Fusion proteins were serially diluted in M-PBS and incubated on coated/blocked plates 1 h at RT. After washing with PBS-T, plates were incubated with an anti-mouse IgG antibody conjugated to HRP, properly diluted in M-PBS. Plates were washed and peroxidase substrate solution (see above) was added. After 15 min the reaction was stopped with 10% (v:v) sulfuric acid. The absorbance at 490 nm was measured in a microplate reader.

### ELISA to assess the inhibition of the interaction between Fc-fused Wuhan-Hu-1 RBD and human ACE2

Polystyrene 96-well microtitration plates were coated overnight at 4 °C with hACE-hFc at 2.5 μg/mL in 0.1 mol/L carbonate-bicarbonate buffer, pH 9.6. The plates were blocked 1 h at 37 °C with 2% (w:v) milk in PBS-T. Convalescent sera were serially diluted in PBS-T containing 0.2% milk (w:v) and 20 ng/mL of Wuhan-Hu-1 RBD fused to mouse IgG2a Fc, and incubated 1 h at 37 °C. The mixtures were added to the coated/blocked plates and incubated 2 h at 37 °C. The plates were washed with PBS-T, and an anti-mouse IgG Fc conjugated to alkaline phosphatase, properly diluted in PBS-T containing 0.2% milk (w:v), was added and incubated 1 h at 37 °C. After washing again, phosphatase substrate solution (p-nitrophenylphosphate at 1 mg/mL in diethanolamine buffer, pH 9.8) was added and incubated during 30 min at RT. The absorbance at 405 nm was determined with a microplate reader. Inhibition capacity of sera was determined taking into account maximum binding (assessed with the fusion protein diluted in blocking solution without sera). Logarithmic transformation was applied to sample dilutions. The data was fitted to a non-linear curve (log(inhibitor) vs normalized response with variable slope) to determine the half-maximal inhibitory dilutions (ID50) for each serum.

### ELISA to assess conformation sensitivity of RBD recognition by anti-RBD mAbs

Polyvinyl chloride microtitration plates were coated overnight at 4 °C with His-tagged Wuhan-Hu-1 RBD at 5 µg/mL in PBS. After washing with PBS, some wells were sequentially incubated 1 h at RT with DTT (0.1 mol/L in PBS) and iodoacetamide (0.1 mol/L in PBS). Non-treated wells were incubated in parallel with PBS. Plates were blocked 30 min at RT with M-PBS. Anti-RBD mAbs (diluted at 5 µg/mL in M-PBS) were incubated 1 h at RT on coated/blocked plates. After washing with PBS-T, the plates were incubated with an HRP-conjugated anti-mouse IgG antibody, properly diluted in M-PBS, during 1 h at RT. Plates were washed and incubated with peroxidase substrate solution as described above. The absorbance at 490 nm was determined with a microplate reader. Relative reactivities of each mAb against reduced/alkylated RBD (%) were calculated, taking the signal of the same mAb on untreated RBD as the reference (100%) in every case.

### In silico structural analysis of mutated RBD variants

The structures of the RBD/human ACE2 complex and the RBD moiety alone were extracted from the Protein Data Bank (PDB ID: 6M0J^12^). Mutated variants' molecular modeling and relaxation steps were performed using Rosetta^60^. Visualization and analysis of the structures were performed with Pymol. Molecular dynamics simulations of both Wuhan-Hu-1 RBD and its mutated variant Y473A were performed using Gromacs 2022.3^61^. Topologies of the systems were built using the CHARMM-36 forcefield. The proteins were placed in a cubic box, with periodic boundary conditions, filled with TIP3P water molecules. For all simulated systems, it was checked that each atom of the protein was at least at a distance of 1.1 nm from the box borders. Each system was then minimized with the steepest descent algorithm. Next, a relaxation of water molecules and thermalization of the system was run in NVT and NPT environments each for 0.1 ns at 2 fs time-step. The temperature was kept constant at 310.15 K (37ºC). Each simulation proceeded during 50 ns of production run. Three replicate runs were performed for each molecule.

### Identification of positive and negative selection events during RBD sequence evolution

The dataset, which had been constructed as described^62^, was taken from https://github.com/spond/SARS-CoV-2-variation (DOI: https://zenodo.org/badge/latestdoi/378638985). Briefly, sequence data were collected from the GISAID^63^ database daily, and full genome viral sequences from human hosts were extracted and processed by a SARS-CoV-2 analysis pipeline (https://github.com/veg/SARS-CoV-2/). Sequences were mapped to the reference genes, using a codon-aware pipeline. The data was compressed to retain a single copy of each unique haplotype in the gene, and any sequences having more than 0.5% uncalled/unresolved (N) bases were filtered out. All sequences were partitioned into 90-days intervals by sampling dates, and subjected to a series of selection analyses. Maximum likelihood phylogenies on compressed data were reconstructed for each time interval, and gene-by-gene distances were estimated to compute diversity and divergence using TN93 distance. Several HyPhy dN/dS-based selection analyses were run on each gene. The analyses, aimed at identifying sites that may be experiencing selection-driven diversification and restricted to internal branches of the tree, included FEL^64^, and MEME^65^. The above-described dataset was searched to look for sites displaying significant signals of positive selection (MEME/FEL *p* values < 0.05) and also negative selection in the case of FEL, among RBD positions targeted in the phage-based mutational scanning experiments. Search was limited to the period between December 2019 and October 2021. A count of selection events was performed. Each event represents the occurrence of a significant signal of selection within the preceding 3-months period.

### Human samples

The protocol for the collection of sera samples from vaccinated individuals was reviewed and approved by the Clinical Institutional Review Board at the Center of Molecular Immunology. Blood extraction was performed by trained clinical laboratory personal following the guidelines established by Cuban Healthcare authorities. All participants provided written informed consent prior to the collection of samples and the request for information about their vaccination schedules.

### Data analysis

GraphPad Prism V.7.04. was used to construct all graphics, to calculate ID50 for sera tested in inhibition assays and K_D_ app values for phage-displayed RBD/ACE2 binding, and to determine the Spearman correlation coefficient between ID50 values calculated using two different assay formats. Kendall’s Tau correlation coefficient between two different sets of measurements of ACE2 binding ability of RBD mutated variants, was calculated using STATISTICA V.7.0.

### Supplementary Information

Supplementary Figures.

## Data Availability

All data generated and analysed during the current study are included in the published article and its supplementary information file. Any additional information is available from the corresponding author upon request.
