# Supplementary material for: Studying SARS-CoV-2 interactions using phage-displayed receptor binding domain as a model protein
Source: Sci Rep. 2024 Jan 6;14:712. doi: 10.1038/s41598-023-50450-4 (PMC10771503; doi:10.1038/s41598-023-50450-4)
Supplement: Supplementary file 1 — Supplementary Figures. [file 41598_2023_50450_MOESM1_ESM.pdf]

Supplementary information

Studying SARS-CoV-2 interactions using phage-displayed receptor binding domain as a model protein

Beatriz Pérez-Massón[1], Yazmina Quintana-Pérez[1], Yaima Tundidor[1], Dayana Pérez-Martínez[1], Camila Castro-Martínez[1], Mario Pupo-Meriño[2], Ivette Orosa[1], Ernesto Relova-Hernández[1], Rosmery Villegas[2], Osmany Guirola[3], Gertrudis Rojas[1*]

[1]Center of Molecular Immunology, calle 216 esq 15, Apartado 16040, Atabey, Playa, La Habana, CP 11300, Cuba

[2]Universidad de Ciencias Informáticas, La Habana, Carretera a San Antonio de los Baños, km 2 1/2, Torrens, Boyeros, La Habana, CP 19370, Cuba

[3]Center of Genetic Engineering and Biotechnology, Ave 31 e/158 y 190, Cubanacán, Playa, La Habana, CP 11300, Cuba

[*] Correspondence should be addressed to G.R.: grojas@cim.sld.cu

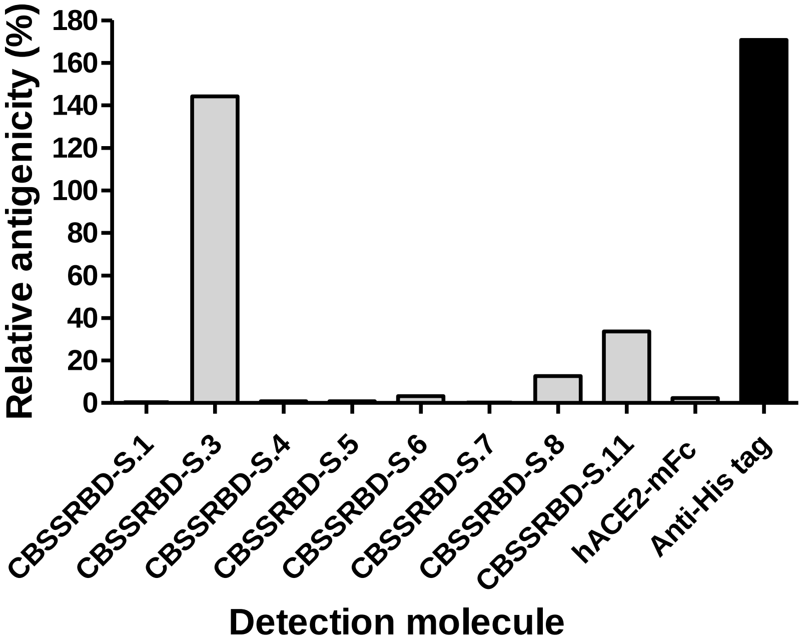

Supplementary figure S1. Conformation-sensitivity of different RBD epitopes. Polyvinyl chloride microtitration plates were coated with a recombinant protein comprising Wuhan-Hu-1 RBD (amino acids 328-533) fused to a C-terminal hexa-His tag. Some coated wells were sequentially treated with the reducing agent dithiothreitol and iodoacetamide. Anti-RBD mAbs were incubated on treated and untreated control wells. A recombinant protein comprising human ACE2 receptor fused to a mouse Fc domain (hACE2-mFc) and an anti-His tag mAb were used as control molecules recognizing conformational and linear epitopes, respectively. Bound molecules were detected with an anti-mouse IgG conjugated to horseradish peroxidase. Recognition of untreated coating RBD by each molecule was used as reference (100%) to calculate relative antigenicity of reduced/alkylated RBD evaluated with the same probe.

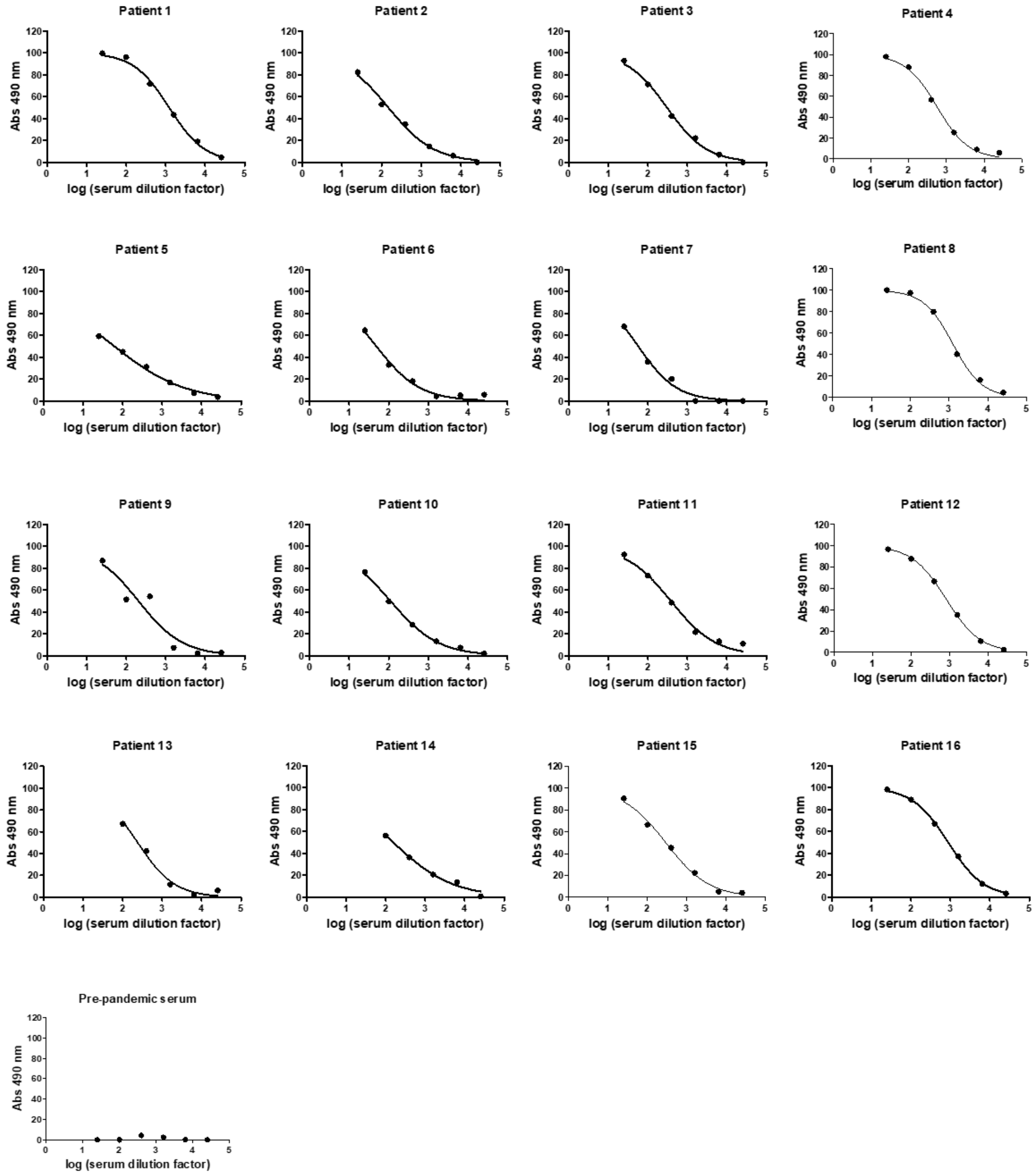

Supplementary figure S2. Inhibition of binding of phage-displayed Wuhan-Hu-1 RBD to immobilized ACE2. Phage-displayed RBD was incubated on polyvinyl chloride microplates coated with human ACE2 extracellular domain fused to human IgG1 Fc. Bound phages were detected with an anti-M13 PVIII antibody conjugated to horseradish peroxidase. Pre-incubation of phage-displayed RBD with serial dilutions of sera from sixteen COVID-19 convalescent patients resulted in dose-dependent binding inhibition. Data were fitted to sigmoidal inhibition curves. Specificity of inhibition was assessed with a sample of pre-pandemic serum (bottom line).

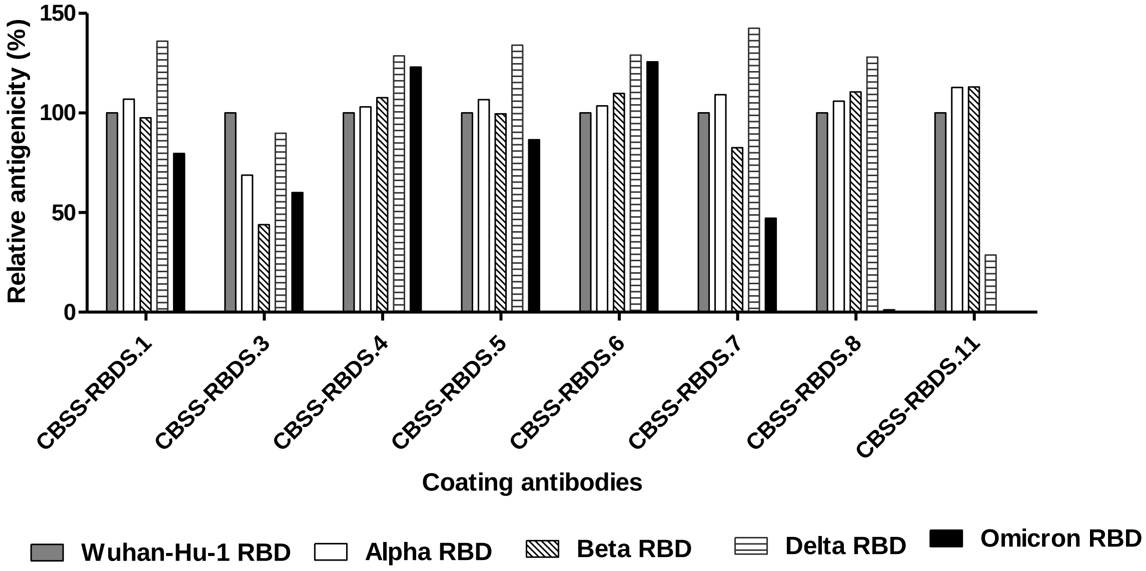

Supplementary figure S3. Antigenicity assessment of phage-displayed RBD derived from SARS-CoV-2 variants of concern. Phages displaying diverse RBD versions, previously normalized according to their reactivity with 9E10 mAb that recognizes the *c-myc* tag fused to all the displayed proteins in our system, were incubated on polyvinyl chloride microplates coated with anti-RBD mAbs (CBSSRBD-S.1-CBSSRBD-S.11). Bound phages were detected with an anti-M13 PVIII mAb conjugated to horseradish peroxidase. Relative reactivities of each variant were calculated taking Wuhan-Hu-1 recognition by the same antibody as reference (100%).

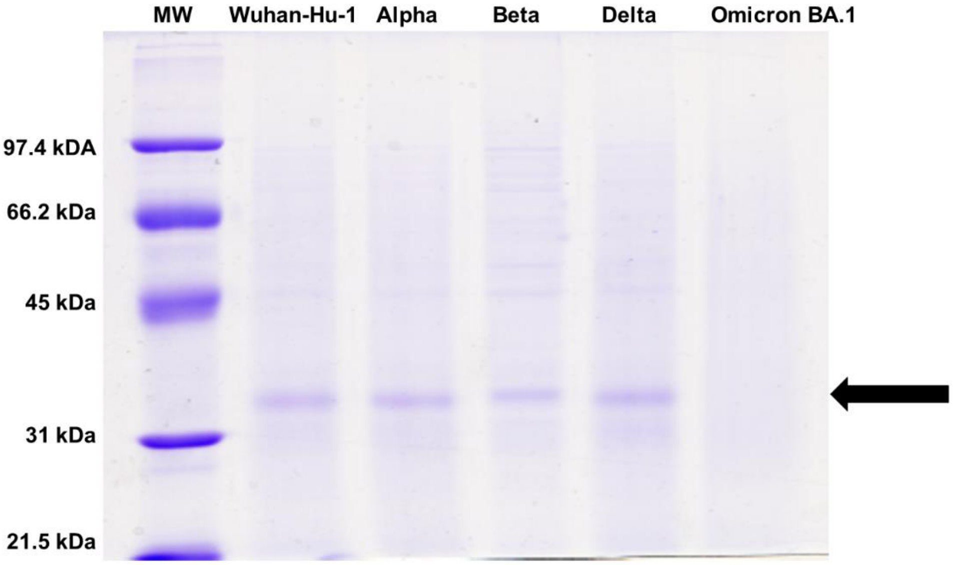

Supplementary figure S4. Production of C-terminal His-tagged RBD variants (aa 328-533) by transiently transfected HEK-293T cells adapted to grow in suspension. Cells were transfected with the corresponding plasmids mixed with polyethyleneimine, and seven days after transfection supernatants were analyzed by SDS/PAGE in 12% gels under reducing conditions. The arrow indicates the position of the band corresponding to RBD variants fused to a C-terminal hexa-His tag. Molecular weight ladder (MW) is included as reference.

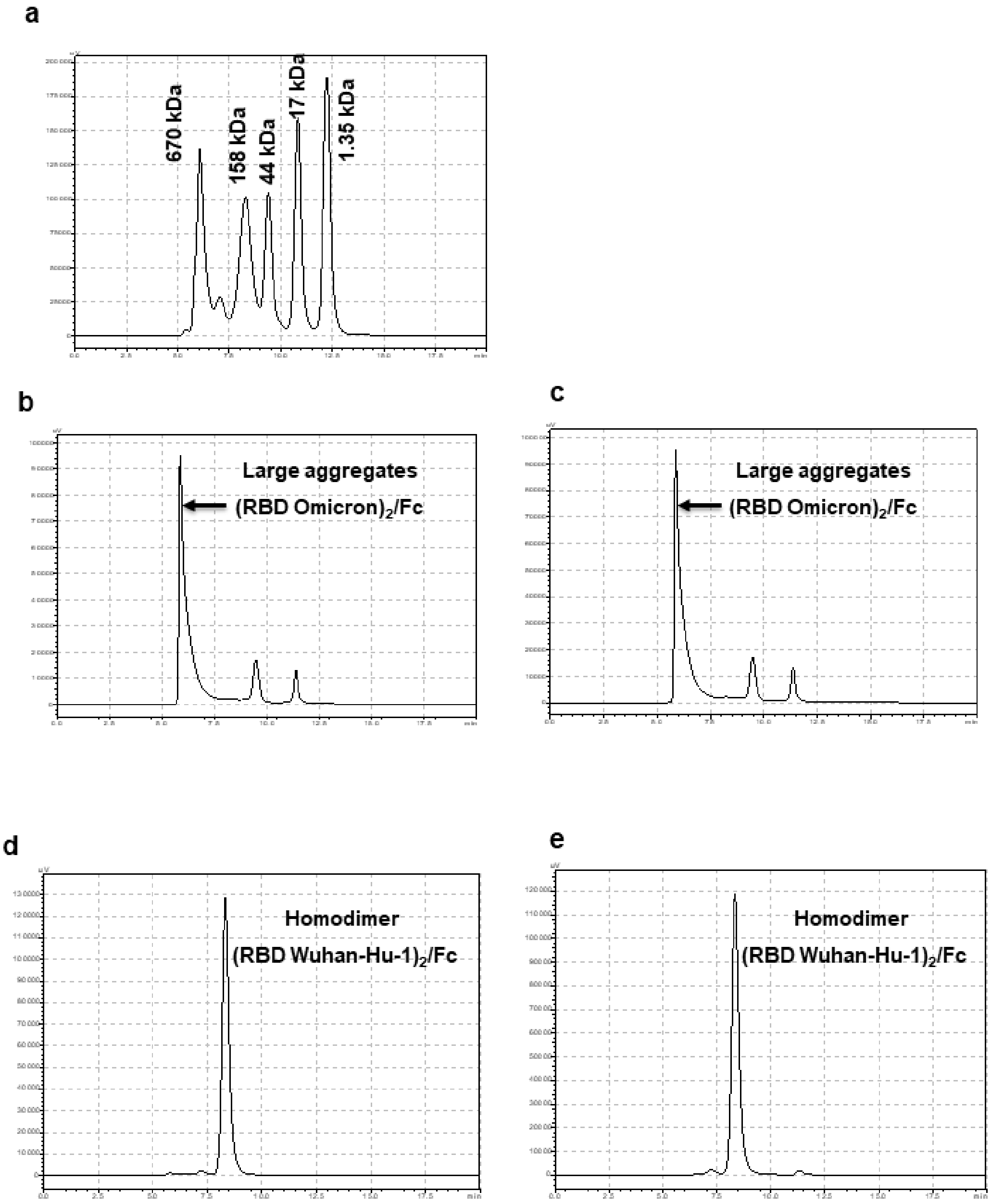

Supplementary figure S5. Aggregation status of recombinant fusion proteins comprising Wuhan-Hu-1 RBD or its mutated Omicron variant and mouse IgG2a Fc. Proteins were obtained after lentiviral transduction of HEK-293 cells with the coding genetic constructs, growth of selected clones in serum-free medium and purification by Protein A affinity chromatography. Purified proteins were analyzed by size exclusion chromatography in a TSKgelG3000SWXL column. Elution profiles of two batches of the fusion protein comprising Omicron RBD (**b-c**) show heavily aggregated products (indicated by arrows), while two batches of Wuhan-Hu-1 RBD-derived fusion protein (**d-e**) exhibit a major peak corresponding to non-aggregated Fc-mediated homodimers. A calibrator comprising five molecules of known molecular weight (Thyroglobulin 670 kDa, IgG 158 kDa, Ovalbumin 44 kDa, Myoglobin 17 kDa, B12 1.35 kDa) was analyzed in parallel (a).

Mutation	ID50	ER*	Mutation	ID50	ER*	Mutation	ID50	ER*
K417A	2029	0.5	Q474K	1403	0.3	F490S	2389	0.4
K417N	1061	1.0	Q474D	1393	0.3	F490D	557	1.8
K417T	402.8	2.7	A475S	1391	1.0	F490V	837.1	1.8
K417R	1830	0.6	A475V	1922	0.7	F490E	826.3	1.2
V445P	1512	0.9	S477N	345.3	2.6	F490L	782	2.0
V445I	1190	1.1	S477P	390	2.3	F490T	3344	0.3
V445K	1089	1.2	S477D	366.8	2.5	Q493S	1636	0.5
V445R	1136	1.2	S477T	646.3	1.4	Q493K	1194	0.7
V445Q	2011	0.8	T478D	992.4	0.7	Q493H	1812	0.5
V445D	3087	0.5	T478V	974	0.7	Q493N	494	1.7
V445S	1565	1.0	T478S	933.1	0.8	S494K	326.6	3.2
V445E	2572	0.6	T478N	750.7	0.9	S494R	272.8	3.8
V445F	2244	0.9	T478K	502.9	1.4	S494A	807.3	1.3
V445W	2460	0.5	T478M	539.1	1.3	S494P	460	3.0
Y449R	1502	0.6	T478R	767.2	1.0	S494N	773.8	1.4
Y449K	1302	0.7	T478A	1158	0.6	S494T	1098	1.0
Y449D	15078	0.1	T478Y	1115	0.7	Q498H	347.7	5.2
L452R	2285	0.8	T478W	1089	0.7	Q498Y	1072	1.7
L452S	2907	0.6	V483R	671	1.8	Q498R	1074	1.7
L452G	1667	1.0	V483A	552.3	2.6	T500A	5758	0.2
L452W	2468	0.7	V483N	620.4	2.0	T500K	3887	0.4
L452I	720.2	2.4	V483S	704.2	2.1	N501Y	75.86	8.5
Y453F	194	8.4	V483D	366.2	3.4	N501T	170.8	3.8
Y453L	587.4	2.8	V483T	641.8	2.3	N501V	271.4	2.4
Y453W	731.3	2.2	E484Q	1239	1.6	N501H	2893	0.2
Y453H	363	4.5	E484T	523	2.4	N501Q	7755	0.1
L455M	1681	0.5	E484A	384.4	3.2	V503H	968.2	0.8
L455T	1904	0.5	E484K	815.2	1.5	V503L	632.4	1.2
L455H	2004	0.5	F486P	2417	0.6	V503A	1429	0.5
L455V	11575	0.1	F486R	1192	1.3	V503S	2026	0.4
L455Q	51366	0.0	F486H	1249	1.2	V503R	1442	0.5
F456L	2959	0.4	F486L	1913	0.8	V503K	1590	0.5
F456V	1878	0.7	F486I	401.7	3.8	V503F	1225	0.6
T470K	1405	0.4	F486V	2031	0.8	V503P	2253	0.3
T470E	2475	0.2	F486N	1683	0.9	Y505W	632.3	1.6
T470H	478	1.0	F486T	2100	0.7			
T470R	1326	0.4	F486G	3097	0.5			
T470L	714.4	0.7	F490K	171.4	8.9			
Y473H	839.7	0.5	F490Y	1062	1.4			
Y473W	2803	0.2	F490P	284.4	5.4			
Q474R	1069	0.4	F490R	956.4	1.0			

Supplementary Table S1. Effect of RBD mutations on inhibition of ACE2 binding by human sera. A pool of sera from 160 individuals who had received a heterologous vaccination scheme, including two doses of SOBERANA 02 and one dose of SOBERANA Plus, was prepared. The capacity of this pool to inhibit the interaction between phage-displayed single-mutated RBD variants and ACE2 was assessed by ELISA. Sub-saturant amounts of normalized phage-displayed mutated RBDs were pre-incubated with serial dilutions of the pool of sera. Similarly diluted phage samples were used as reference (100% binding). Samples were incubated

on polyvinyl chloride microplates coated with the recombinant fusion protein comprising the extracellular domain of human ACE2 fused to a human IgG1 Fc domain. Bound phages were detected with an anti-M13 PVIII antibody conjugated to horseradish peroxidase. Data were fitted to sigmoidal inhibition curves, and half-maximal inhibitory sera dilutions (ID50) were estimated. The escape ratio (ER) was defined as the ratio between the ID50 value determined for the original phage-displayed Wuhan-Hu-1 RBD (*) and the one calculated for each mutated RBD. An escape ratio (ER) value >1 indicates a diminished ability of sera to inhibit binding of a given variant, while ER ≤1 corresponds to equal or higher sera inhibition capacity as compared to the original RBD.

(*) Since the calculated values of ID50 for phage-displayed Wuhan-Hu-1 RBD showed inter-assay variability (ID50=1150±418.8 in the whole set of 32 independent experiments), ER for a given mutated variant was calculated taking into account the reference Wuhan-Hu-1 ID50 determined in parallel in the same experiment where the variant of interest was evaluated.

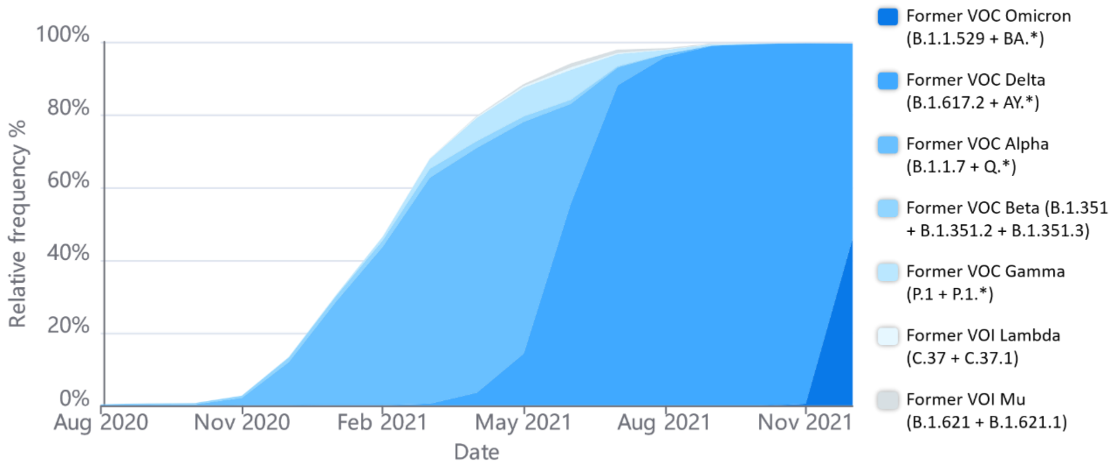

Supplementary figure S6. Relative abundance of different SARS-CoV-2 variants during COVID-19 pandemic. New mutated variants derived from the original Wuhan-Hu-1 emerged at different time points. While some of them remained as minor contributors to the global viral spreading, the frequency of others increased sharply, giving rise to several infection waves characterized by the predominance of a particular variant (identified with color codes). Data were extracted from GISAID database.
